# How close have historical domestication and modern breeding come to ideotypes for yield?

**DOI:** 10.1016/j.plaphe.2026.100290

**Published:** 2026-10-10

**Authors:** Yixiang Shan, Jochem Evers, Bo Sun, Colin P. Osborne

**Affiliations:** a Plants, Photosynthesis and Soil, School of Biosciences, University of Sheffield, Sheffield, S10 2TN, UK; b Institute of Emerging Agricultural Technology, Shenzhen University of Advanced Technology, Shenzhen, Guangdong, China; c Centre for Crop Systems Analysis, Wageningen University and Research, Wageningen, the Netherlands; d Department of Automatic Control and Systems Engineering, University of Sheffield, Sheffield, UK

**Keywords:** Wheat, Domestication, Ideotype, Functional-structural plant (FSP) model, Plant evolution

## Abstract

Optimizing crop architecture and allocation of resources to grain yield have been central goals in cereal breeding. Whether historical selection has achieved an ideotype maximizing yield under modern cultivation remains unclear. In this study, we explored how morphological traits have changed in wheat (*Triticum aestivum*) and whether they approach predicted optima for yield and resource allocation, using functional-structural plant (FSP) modelling. We used phenotypic data from a diverse panel of 142 wild, traditional and modern wheat accessions to parameterise and calibrate a functional-structural plant model. The model predicted which combinations of architectural and allocation trait values conferred maximal grain production under contrasting planting densities representing historical extensive and modern intensive agriculture. Our results show that historical changes to crop traits during domestication and landrace selection have converged on the ideotype by increasing the values of spike, internode and leaf carbon sink capacities. However, the specific internode length and leaf insertion angle became smaller following domestication, while the historical ideotype suggested these traits should increase. Under high planting density, the optimal values of these two traits would also be larger in modern ideotypes. Modern wheats have approached the ideotype by increasing potential spike biomass and decreasing the potential biomass of internode and leaf, but crop architecture could be improved. Historical selection has optimised biomass allocation for landrace cultivation under low densities. Allocation in modern varieties is also optimal for high-density modern agriculture, but further selection on architecture may increase yield potential.

## Highlights

• Integrating functional-structural plant modeling with natural variation identified optimal ideotypes for wheat yield.
• Historical selection aligns with yield-optimised traits at low planting density, reaching theoretical optima for allocation.
• Modern cultivars possess potential for improvement at high planting density by modifying leaf biomass and plant architecture.

## Graphical abstract

**Figure:**
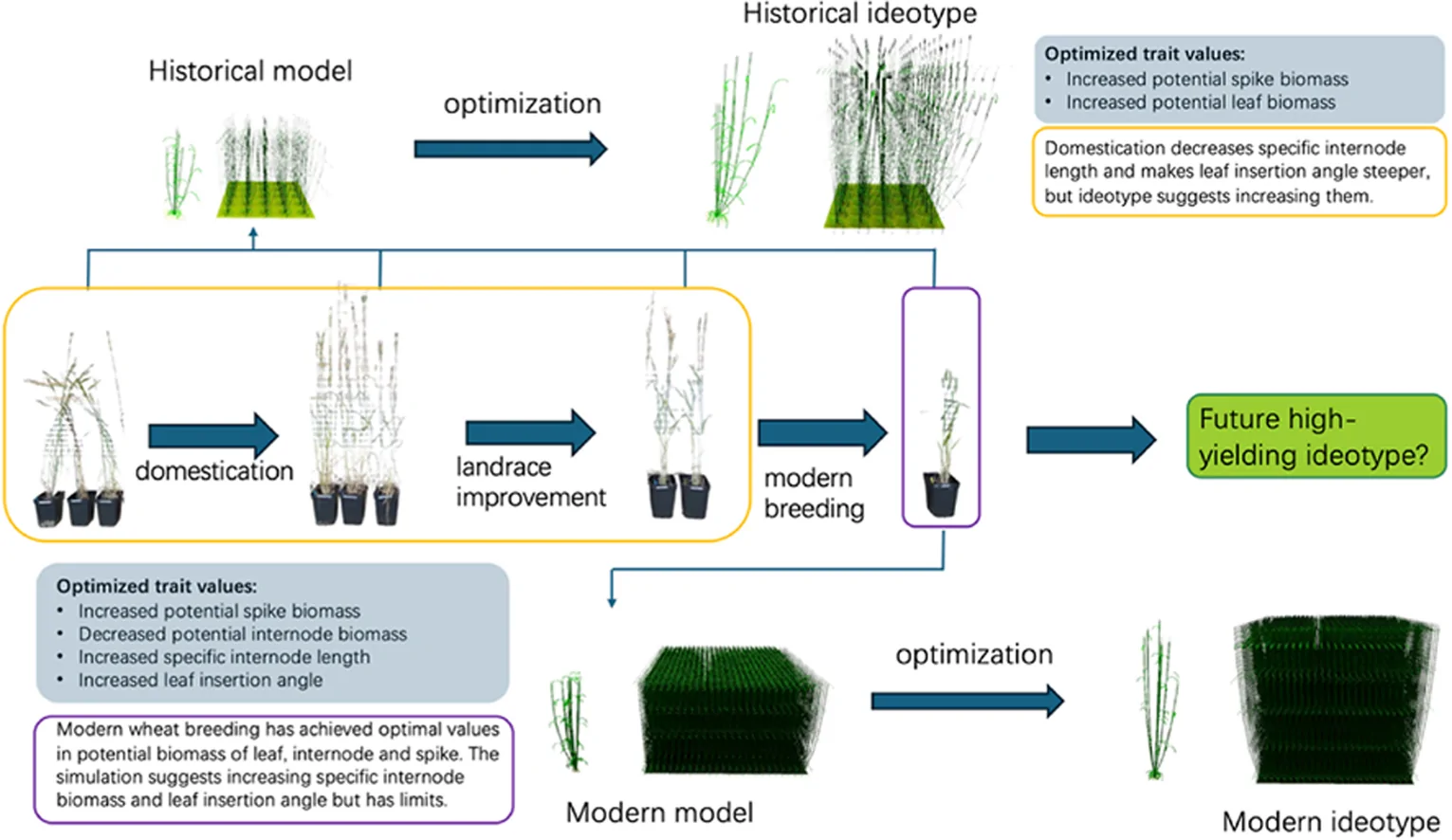

## 1 Introduction

Population growth, the dietary transition and climate change necessitate a 70% increase in global crop production by 2050 to ensure food security [1]. Among various strategies to enhance crop yield, optimizing plant phenotypes has emerged as an effective approach [2]. This strategy seeks to change phenotypic trait values to achieve desired functional outcomes [3], [4]. The idea of combining multiple phenotypic traits to improve crop performance can be traced back to Donald's concept of “ideotype breeding” [5]. This method defines target traits based on breeding objectives, and then designs optimised combinations of trait values to construct an ideotype that can achieve those objectives. Advances in computational modelling have enabled researchers to simulate and optimise plant traits to develop ideotypes, as demonstrated by numerous examples such as optimizing flag leaf area using the *Sirius* model [6], maximum root length with the *SAMARA* model [7], source-sink strength using the *Greenlab* model [8], and flowering time and leaf size with the *CSM-CROPGRO-Peanut* model [9].

Prior to the formalization of ideotype breeding in the 1960s, historical selection through crop domestication and breeding had already driven substantial phenotypic evolution. In wheat (*Triticum spp.*) major yield improvements have historically resulted from domestication, landrace development, and the Green Revolution [10], [11]. These processes have been accompanied by selection on key phenotypic traits such as spike number [12], spike weight [13] and leaf insertion angle [14]. The Green Revolution particularly achieved substantial gains in yield by reducing plant height and reallocating biomass from stem and leaves to the spike [15]. However, the extent to which historical phenotypes has converged on theoretical ideotypes has not yet been systematically evaluated. Evaluating this gap between past selection outcomes and optimal ideotypes is particularly important now, since modern breeding increasingly relies on wild germplasm to introduce novel diversity and overcome the genetic bottleneck present in modern elite varieties [16]. For instance, selective breeding has directly targeted morphological traits like shorter stems to achieve agronomic benefits such as lodging resistance [15]. Other changes, such as steeper leaf angles, were selected as passive adaptations to agronomic conditions such as planting density and fertilization [17]. Recognizing how these past selection pressures shaped plant architecture highlights the potential to further exploit underutilized traits, such as grain sink capacity, in modern ideotype breeding.

Crop traits have been evolving continuously since the earliest cultivation via a long-term process [18]. The phenotypic evolution of wheat has followed several stages, each characterised by different multiple selection pressures [19]. In the first stage, the polyploidisation of wild wheat increased leaf biomass and grain weight, but reduced tillering [19]. Domestication then occurred, bringing the potential for both directed selection by farmers and natural selection. The natural selection pressures on early domesticated crops stemmed from the transition from natural ecosystems to cultivated fields, driving the emergence of phenotypic trait values adapted to the new growing environment [17]. It is widely believed that early agricultural lands were resource-rich, favouring low-density cultivation, and archaeobotanical evidence suggests that plant densities in the earliest cultivated plots were strikingly low by later standards. Bogaard shows that Neolithic weed assemblages from central Europe cluster around disturbance-tolerant annuals typical of small, hand-tilled beds rather than the dense, uniform stands produced by later plough agriculture [20]. Likewise, Halstead reports modern Mediterranean farmers broadcasting only 100–150 kg of wheat seed per hectare on thin, sloping soils [21]; when projected onto the lightly manured, frequently fallowed plots of the Neolithic, this implies establishment densities well below 50 plants m^−2^, a figure consistent with the sparse, patchy crops inferred from early weed floras [22]. Evidence for crops grown using traditional cultivation practices showed that the yield in 1940 was only 0.6 t/ha [23] far lower than the 7.3 t/ha in the UK in 2024 [24]. Historically cultivated crops thus evolved under low-density conditions, with planting densities increasing over time alongside advances in intensive agriculture [25], [26]. Subsequent landrace improvements reduced tillering and spike numbers, concentrating resources on thicker main stems and larger flag leaves [27]. Subsequently, modern wheat emerged following the Green Revolution of the 1950s and 1960s, undergoing further selective breeding to the present day [28]. Considering the trait values derived from all wheat varieties that have appeared throughout history, is the optimal combination of these trait values embodied in today's wheat? **This leads to our first research question: have domestication and historical landrace evolution favoured trait combinations that maximised yield under low-density planting conditions?**

The Green Revolution took place between 1950 and 1960 [10]. During this period, the values of some previously selected trait values reversed direction [29]. For example, traditional tall-stemmed varieties were replaced by short-stemmed varieties. Similarly, the decline in reproductive allocation observed during domestication reversed in modern varieties [19]. However, it remains unclear whether these reversals have approached the theoretical optimum of high-density planting systems or are still in progress. Addressing this uncertainty is crucial because deviations from the optimal phenotypic space mean that opportunities for further genetic improvement or management interventions have not yet been realized. Currently, planting density has increased to 400 plants m^-^^2^ [30], although dense canopies may lead to higher disease susceptibility [31]. Donald [5] noted that the ideotype optimised for individual performance may become less suitable under high-density planting due to resource competition, a view supported by modern agroecological theory [32]. This arises because there is a trade-off between individual competitiveness and a population-friendly phenotype: individual plants with strong competitiveness gain an advantage by capturing limited resources, but this inhibits the growth of neighbouring plants, while group-friendly traits emphasise the equitable distribution of resources to increase overall yield [33]. Recent studies show that wheat competitiveness increased during domestication but decreased in modern breeding [17]. Modern breeding has therefore acted to reset the crop towards population-friendly traits from individual competitiveness. However, whether group-friendly varieties have reached their theoretical optimum remains unclear. **This raises our second research question: has modern wheat breeding already achieved the theoretical ideotype for current high density crops?** If modern wheats have not yet reached their theoretically ideotype state under high density planting, it means that there is still potential for further genetic improvement or management intervention.

To answer the questions above, we need to select an appropriate modelling method. APSIM-Wheat [34] and DSSAT-CERES-Wheat [35] simulate phenology, biomass accumulation, soil water and nitrogen dynamics, and yield across environments and management regimes. CN-Wheat represents carbon and nitrogen acquisition and allocation among wheat organs after anthesis [36]. These models simulate wheat growth across organ, plant, canopy and field scales, but most do not explicitly combine three-dimensional organ geometry, local light interception and competition among neighbouring plants needed for multidimensional optimization of architecture and biomass allocation. A spatially explicit Functional Structural Plant (FSP) model [37] is necessary to test how architecture and biomass allocation affect yield at contrasting planting densities. In this study, we aimed to test whether trait evolution during past domestication and modern breeding aligns with ideotype predictions. Using phenotypic data from a previous experiment [19], we calibrated two FSP models. The first was parameterized using average trait values from wild ancestors, domesticated wheat, landraces and modern wheat, representing a “historical wheat” baseline. Optimizing architecture and allocation at low planting density in this model allowed us to explore whether domestication outcomes have mirrored model-predicted optima. The second model used trait values from modern wheat cultivars, enabling us to evaluate the potential for further improvement through the optimization of architecture and allocation under high planting density. After model calibration, we systematically altered trait values to identify ideotypes with improved yield.

## 2 Methods

### 2.1 Modelling approach

We used an FSP model to optimise ideotypes for yield within the constraints of empirically measured phenotypes. FSP models are computational models that integrate three-dimensional (3D) plant structure (morphology) with its physiological functions such as photosynthesis, transpiration, and water transport [38]. They simulate the dynamic growth processes of individual plants within their environment and are frequently employed to study plant responses to factors including light, nutrients, and pruning [39]. The optimization was carried out using a model based on principles described in Ref. [40] created previously within the GroIMP platform (www.gitlab.com/grogra/groimp). The updated model used in this work is at the repository (https://github.com/YixiangShan/FSP_wheat_domestication)

The phenotypic data came from previous work [19], in which morphological traits of 142 wheat accessions were measured, spanning the diversity of wild, domesticated and improved accessions. These empirical observations were used to constrain the modelling in three ways. First, we used average morphological phenotypes as starting points for the optimization process (i.e. the baselines for our simulations). Second, we optimised a selection of morphological model parameters within the ranges observed in a diverse panel of historical and modern plant accessions. The five selected traits were measured directly or calculated from measured traits and were used as model inputs, not model outputs. Third, to account for trade-offs among plant traits, we used a convex hull approach to further constrain the model parameters, allowing only the combinations of values that were observed in the diversity panel. This conservative constraint was intentional because our aim was to identify yield optimizing combinations supported by observed wheat diversity, rather than to predict combinations beyond the current germplasm panel.

Five model parameters were selected to optimise. All of these parameters rank highly in FSP model yield sensitivity analysis [41] and change significantly during domestication [19]. They are [wmaxFlower], [wmaxLeaf], [wmaxInt], [specificInternodelength], and [leafAngle] (which concurrently controls both upper and lower leaf insertion angles in the model). In this work, the first four parameters are described as follows: “potential spike biomass”, “potential leaf biomass”, “potential internode biomass” and “specific internode length”. The final parameter is referred to as “leaf insertion angle”. To facilitate reader comprehension, these parameters are described using these biological terms, and the specific correspondences to model parameters can be found in Table S1.

We carried out two model optimisations as shown in Fig. 1. The first optimization, termed historical wheat, started from a baseline derived from average parameter values across all of the wild, historical and modern wheat varieties in our dataset. The model was set up to simulate a low planting density corresponding to historical and pre-industrial cultivation practices, and the parameters were optimised in order to maximize plant individual yield. We then compared the optimised parameter values with those observed in wild, historical and modern varieties. The second optimization started from a baseline derived from modern wheat parameter values and simulated a high planting density typical of modern intensive industrial agricultural practices. We compared the optimised parameters with the observed values in modern varieties.

**Fig. 1:**
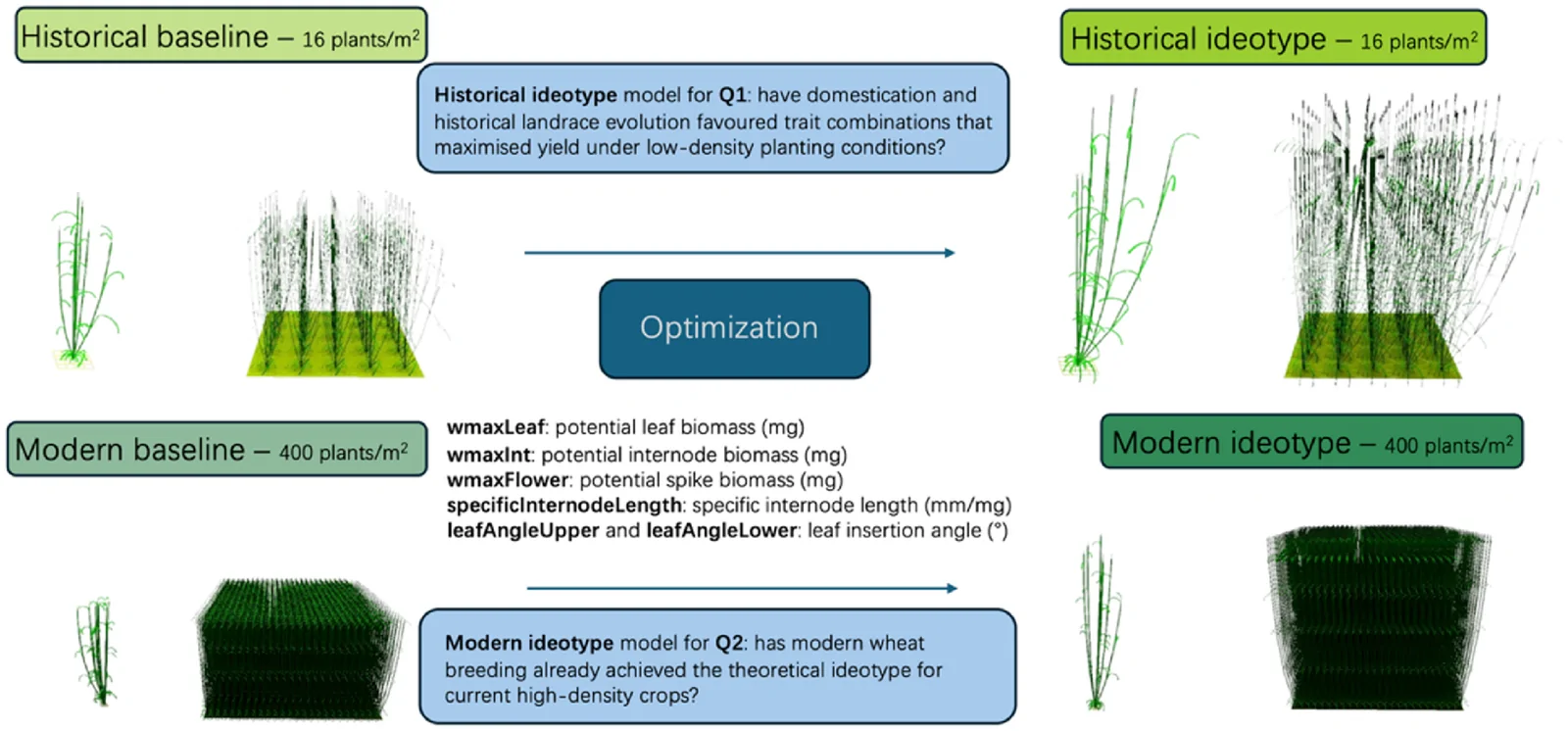
The optimization process applied using the FSP model. Schematic overview of the two central research questions and modelling workflow used to compare “yield-optimised” ideotypes with wheat evolutionary history and modern breeding. Q1: Historical ideotype vs. domestication. A baseline FSP model was parameterized using mean trait values from eleven historical wheat taxa (wild diploids, tetraploid landraces and early *T. aestivum*) and run at a density of 16 plants/m^2^. Q2: Modern ideotype vs. intensive breeding. A second FSP model baseline was built from trait means of 21 modern *T. aestivum* varieties and run at 400 plants/m^2^. The traits for optimization are potential leaf biomass, potential internode biomass, potential spike biomass, specific internode length and leaf insertion angle.

### 2.2 Model development

We first classified the morphological data according to species, and then calculated mean trait values for each. We then used these values to calibrate the FSP model for each species (Table S1). In our previous experiments, wheat plants were arranged in 5 × 5 blocks (25 plants per block) with both plant distance and row distance of 0.25 m, corresponding to a planting density of 16 plants/m^2^. We first selected FSP model parameters that we measured directly in our experiment (Table S2), and ran the model to produce a phenotype for each species at the experimental density of 16 plants/m^2^. We then tuned the apical dominance parameter, keeping the values of other parameters unchanged, until height and tiller numbers approached the observed values (within one standard error) for each species. Tiller number in the model is not prescribed directly but emerges from the interaction between apical dominance and the source–sink balance. Density effects are therefore represented indirectly through their influence on plant spacing, local light interception and resource availability rather than through an explicit density-dependent tillering rule. Data not obtained from our experiments have been set to the model's default values for wheat (Table S1). We then conducted an internal evaluation by comparing outputs from the historical and modern baseline models at 16 plants/m^2^ with the corresponding observations. For grain yield per plant, aboveground biomass, stem biomass, leaf biomass, plant height and spike number, we calculated the observed and simulated means and standard deviations, the mean difference, and the absolute difference relative to the observed standard deviation (Table S3). Because the same dataset informed model parameterisation and calibration, this analysis assesses agreement under the calibration conditions rather than independent predictive performance. For all six outputs, the simulated means were within one observed standard deviation of the corresponding observed means, with absolute differences of 0.04 to 0.42 standard deviations for the historical baseline and 0.06 to 0.94 standard deviations for the modern baseline.

We next parameterized baseline cases for a historical wheat and a modern wheat (Fig. 1, Q1 and Q2). These two models served as the starting points for optimization and were shown in Table 1. The historical model was produced using the average trait values for wild *T. urartu*, wild *T. boeoticum*, wild *T. araraticum*, wild *T. dicoccoides*, domesticated *T. dicoccum*, domesticated *T. timopheevii*, domesticated *T. monococcum*, landrace *T. durum*, landrace *T. aestivum,* modern *T. durum and* modern *T. aestivum*. The phenotype coming from this simulation is called “historical baseline” The modern model was parameterised using trait averages for modern *T. aestivum* with varietal release dates spanning the 20^th^ and 21^st^ centuries [19]. The phenotype produced by it is called “modern baseline”. The historical baseline and modern baseline represent FSP virtual plants before optimization.

**Table 1:**
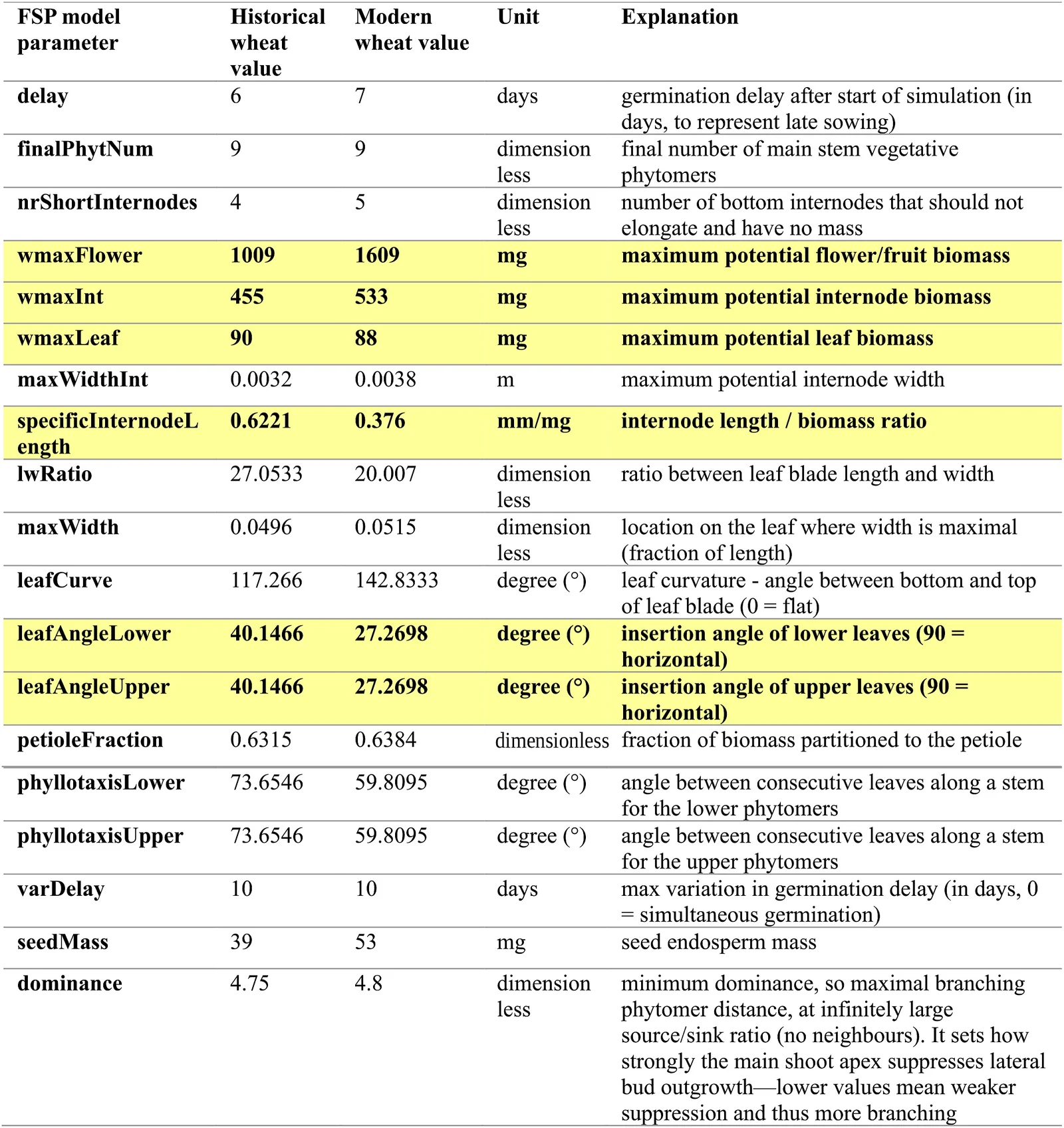
**FSP model parameter values used for historical and modern wheat baselines.** All values are given to the precision required by the model. The parameter with yellow colours are the ones we optimised.

For optimization simulations, we set the modelled density at 16 plants/m^2^ for the historical ideotype, and 400 plants/m^2^ for modern ideotype respectively. The setting of 400 plants/m^2^ was chosen to simulate the optimization of the modern ideotype under high density conditions. After the trait optimization for the modern ideotype were completed, we returned the generated modern ideotype to a density of 16 plants/m^2^ for a further growth simulation, thereby generating output values that could be fairly compared with empirical data for modern wheat from our previous domestication work [19].

### 2.3 Trait selection and filtering

Five traits mentioned above were selected for optimization (Table 1). Before optimizing trait values within the model, we first needed to account for correlations among traits that indicate trade-offs to avoid creating unrealistic plants [42]. For example, optimization cannot pick the largest flower biomass and the smallest internode biomass, since this trait combination would mean that shoots are too mechanically weak to support heavy spikes; a biomechanical element the model does not simulate. To account for such limitations, we applied a convex hull algorithm to filter out only those combinations of trait value ranges that were observed across the diversity of wheat accessions surveyed [43]. We then only considered these trait values in our optimization procedure.

To ensure the biological plausibility of the simulated phenotypic combinations, a five-dimensional (5D) convex hull was constructed based on the experimental data for the five traits (potential spike biomass, potential leaf biomass, potential internode biomass, specific internode length, and leaf insertion angle). A hyperdimensional grid was generated by dividing the observed range of each trait into ten equal intervals. To maintain computational feasibility and biological realism, only the grid points residing within the 5D convex hull were retained for FSP model simulations (to visualise this concept, a 3D projection using potential biomass of spike, internode, and leaf, is presented in Fig. S1). For each trait, we chose the maximum and minimum values and divided the range of values between them into ten equal parts. After that, we used permutation and combination on ten values of flower, internode and leaf biomass to form a three digit matrix of 10∗10∗10 trait combinations (Fig. S1D) and in the 5D convex hull there are 10^5^ combinations. The matrix was then filtered using the convex hull (Fig. S1E). The points outside the convex hull were excluded as being unfeasible in reality, and the points inside were retained as having trait-trait relationships within the envelope of natural variation (Fig. S1F).

Next, we input all the retained data points into the FSP model baseline and simulated growth for each combination. At this step, the 5D convex hull actually has 1598 data points of combination. We recorded the combination of traits producing the highest yield among the model outputs. The corresponding trait values represent the optimised wheat ideotype, which were recorded as the historical ideotype and modern ideotype respectively. Besides the ideotype, which ranks first in yield, we also found that the yield differences among the top 1% of wheat varieties were very small. Therefore, we selected 16 simulation combinations from the top 1% of yield in both the historical and modern model simulations as the top 1% ideotype candidates.

### 2.4 Historical ideotype data analysis

In the results for the top 1% historical ideotype candidates, principal component analysis (PCA) was used to reduce the dimensionality of the multidimensional trait data. First, based on the mean and standard deviation of the real wheat dataset, Z-score standardization was performed on the five core traits (potential spike biomass, potential leaf biomass, potential internode biomass, specific internode length, and leaf insertion angle) to eliminate the influence of different physical dimensions on spatial distance calculations. Subsequently, the standardized simulated and real data were projected onto a two-dimensional coordinate system constructed using the first two principal components. To identify ideal phenotypic regions, the geometric boundaries of the top 1% historical ideotype candidates in the simulated data were calculated, constructing a high-yield phenotypic convex hull. Simultaneously, the loading matrices of each trait were superimposed as vectors in the PCA space to clarify the contribution intensity and direction of each phenotypic parameter to the spatial distribution.

To test whether the historical ideotype in each case changed the traits compared with the baseline cases and real wheat, we applied ANOVA analysis with a Tukey-HSD test. In these simulations, each historical ideotype was represented by a population of 25 individual wheat plants arranged in a 5 × 5 grid. These 25 individuals served as the replicates for all statistical tests to account for individual plant differences.

To identify the core phenotypic factors driving wheat yield and evaluate the predictive efficacy of the FSP model, feature importance analysis was performed using a Random Forest regression algorithm (the number of trees was 500). Yield prediction models were constructed for both real wheat data and FSP model output data. Permutation importance was used as a metric to quantify the contribution of each phenotypic trait to yield variation. To enhance comparability across different datasets, the original importance values of each trait were scaled to a maximum of 100 within each dataset, with negative values retained.

### 2.5 Modern ideotype data analysis

In the analysis of the modern ideotype, we also made comparisons using general linear modelling of observed modern wheat traits in relation to the release date (1935 - 2006) for each variety. This linear model was then used to compare the y-axis trait values for modern wheat to determine whether they have obtained ideotype values.

Our observed phenotypic data for modern wheat are based on an experimental planting density of 16 plants/m^2^, such that we lack real world data for modern wheat at 400 plants/m^2^. Therefore, we can only compare the input values (the five optimised trait values) obtained from optimizing the modern ideotype at 400 plants/m^2^ with those of modern wheat at 16 plants/m^2^. Conversely, we cannot compare the model output values of the modern ideotype at 400 plants/m^2^ with those of modern wheat at 16 plants/m^2^. We therefore quantify the optimization results by comparing the modern baseline (i.e., the model based on the measured phenotypic traits of real modern wheat) at 400 plants/m^2^ with the optimised modern ideotype at 400 plants/m^2^.

To quantify the minimum trait adjustment required for real modern wheat to evolve towards the top 1% modern ideotype candidates, we employed the Nearest-Neighbour Mapping method in five-dimensional trait space. For each real wheat sample, the Euclidean distance between it and all points within the simulated high-yield region of top 1% ideotype candidates was calculated in the standardized 5D trait space and mapped to the nearest target ideotype. Thus, this step is ensuring the minimum biological cost of the trait improvement path. Subsequently, the incremental migration of each sample from the observed value to the target value in each trait dimension was calculated and restored to the original physical units. The statistical distribution of the trait migration across the entire population was displayed using a combination of violin plots and box plots, thereby identifying the core phenotypic gaps restricting further yield improvement in current wheat varieties and their improvement directions.

## 3 Results

### 3.1 Historical ideotype results

The historical ideotype and all the 1598 simulated combinations are shown in Fig. S2A. The highest yielding historical ideotype did not reach the observed minimum or maximum of any single trait, but its distance to the five dimensional hull surface was in the lowest 12.6% of all feasible combinations. Among the 16 top 1% candidates, 11 were in the lowest 25% of boundary distances, including five in the lowest 10%. High yielding candidates therefore tended to occupy uncommon regions near the hull boundary, although some remained further inside the feasible space. The optimised values of five parameters and original values are listed in Table S4. The real wheat data and the optimization data were combined and the overall visualization is shown in Fig. 2. Under the convex hull constraint, the model's data points (green) all fall within the area of the real wheat (yellow) morphospace (Fig. 2). We found that high yielding model data points appear in the lower right part of the PCA, i.e., those with higher potential spike biomass, potential leaf biomass, and potential internode biomass. In contrast, specific internode length has a more negative effect on high yield. It is worth noting that not all data points within the convex hull encompassing the top 1% historical ideotype candidates are high yielding. A large proportion of data points still have very low yields, even though they are within the top 1% yield convex hull.

**Fig. 2:**
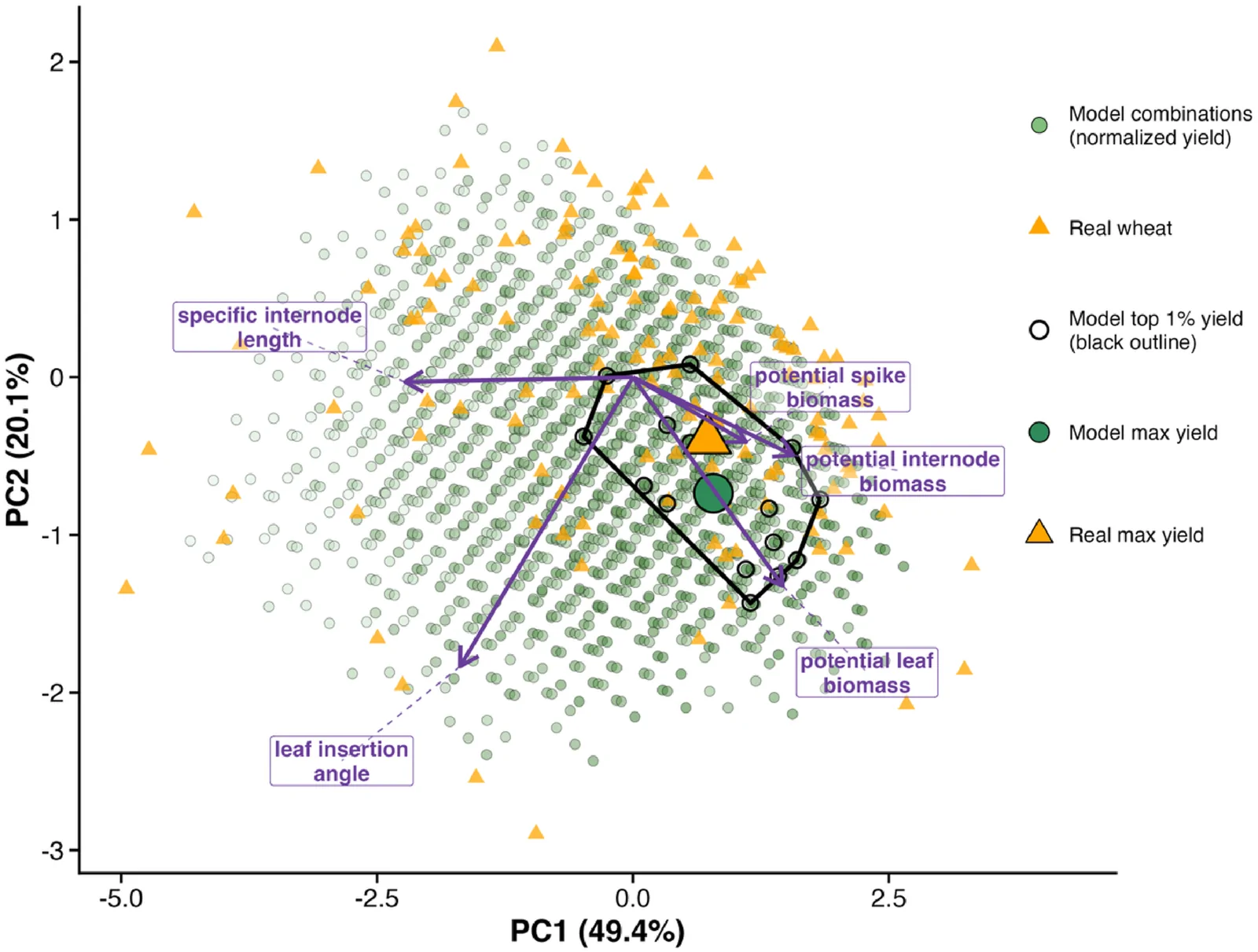
Principal Component Analysis of morphospace comparing simulated model combinations and real wheat performance. The scatter plot represents the spatial distribution of phenotypes mapped based on five key traits: potential spike biomass, potential leaf biomass, potential internode biomass, specific internode length, and leaf insertion angle. Green dots (with gradient background colour) represent different trait combinations simulated by the model, with the shades corresponding to normalized yield (0 to 1); orange triangles represent actual wheat observation data. The convex hull enclosed by the solid black line defines the top 1% of high yield combinations in the simulated output. The highest yield points in the simulated and actual data are marked with large circles and large triangles, respectively.

To test differences among observed wheat groups, we applied one way ANOVA followed by Tukey HSD. Simulated scenarios were excluded from these tests. The 25 plants within each simulated stand share the same parameter set and environment and were therefore treated as individuals within one stand rather than independent replicates. Their distributions were retained to describe variation within the stand. For each simulated trait, we calculated the percentage change from the historical baseline to the best ideotype as a descriptive effect size. The 16 parameter sets producing yields in the top 1% were used to show the range of near optimal solutions and were not included in inferential tests.

The optimised trait value range of the top 1% historical ideotype candidates and the optimised trait value of the most productive historical ideotype show overall consistency. However, there is large variance in some traits such as potential leaf biomass (Fig. 3; Table S4). The five traits selected after optimization with overall yield as the target showed different values. The potential internode biomass and potential leaf biomass both increased from low to high during domestication (Fig. 3B and C). Based on optimization comparisons, the potential leaf biomass in modern *T. aestivum* needs to be increased from its current baseline of 88 mg to reach the optimal target of 138 mg. Conversely, the potential internode biomass in modern *T. aestivum* (534 mg) appears to have reached the recommended optimal values for the historical ideotype (536 mg). The historical ideotype follows the same trend for potential spike biomass, whose value steadily increased during domestication, reaching 1609 mg in modern wheat, close to the best ideotype value of 1684 mg (Fig. 3A). The specific internode length has decreased during wheat evolution, which means plant stems have become shorter, thicker and/or denser (Fig. 3D). The historical ideotype suggests changing this trend to make stems longer per unit of biomass, increasing specific internode length from 0.62 in the historical baseline to 0.81 in the best ideotype (Fig. 3D). Similarly, the leaf insertion angle has also become domesticated to smaller values to make shoot architecture more compact (Fig. 3E). However, the best historical ideotype suggests a reversal in this trend, with the leaf insertion angle increasing to 35.8°, compared to 27.3° for modern *T. aestivum* (Fig. 3E).

**Fig. 3:**
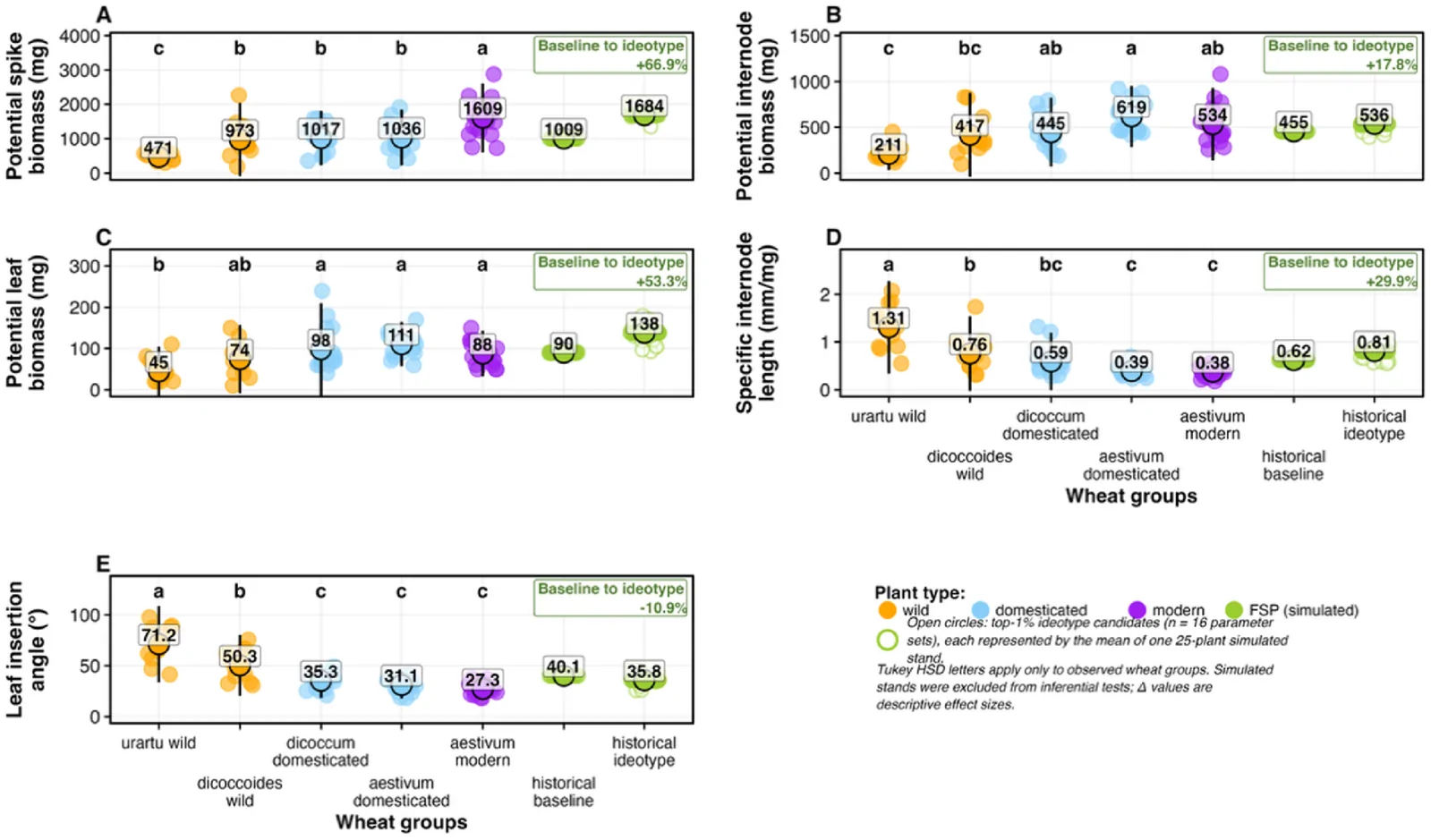
Measured trait values for the historical diversity of wheats compared with the optimised ideotype values derived from the FSP model using the historical baseline. (A) Potential spike biomass; (B) potential internode biomass; (C) potential leaf biomass; (D) specific internode length; and (E) leaf insertion angle. Points represent individual observed accessions. Large points and error bars show observed group means ± SD. The historical baseline and best ideotype are each shown as one value because the input traits were fixed within each simulation scenario. Open green circles represent the top 1% ideotype candidates (n = 16 parameter sets), and each circle represents the mean of one 25 plant simulated stand. Percentage changes from the historical baseline to the best ideotype are descriptive effect sizes. Different letters indicate differences among observed wheat groups at P < 0.05 using Tukey HSD. Simulated scenarios and top 1% candidates were excluded from inferential tests. All optimisations were carried out at 16 plants/m^2^.

Fig. 4 compares the output values of the historical ideotype and historical baseline with those of wheat varieties composed of wild diploid and tetraploid, domesticated tetraploid landraces, hexaploid landraces, and modern improved varieties. Grain yield per plant increased by 91.8% from 4.08 g in the historical baseline to 7.82 g in the best ideotype (Fig. 4A). The best ideotype also had higher mean grain yield than the observed wheat groups, including modern *T. aestivum* at 6.20 g. Aboveground biomass increased by 57.9%, from 14.90 to 23.52 g per plant (Fig. 4B). Stem biomass increased by 37.4%, from 9.01 to 12.38 g per plant (Fig. 4C), and leaf biomass increased by 83.3%, from 1.81 to 3.32 g per plant (Fig. 4D). Plant height increased by 39.5%, from 99.6 to 139.0 cm (Fig. 4E). Spike number changed by only 1.2%, from 6.9 to 7.0 per plant, and remained below the observed mean of 15.8 in wild *T. urartu* (Fig. 4F).

**Fig. 4:**
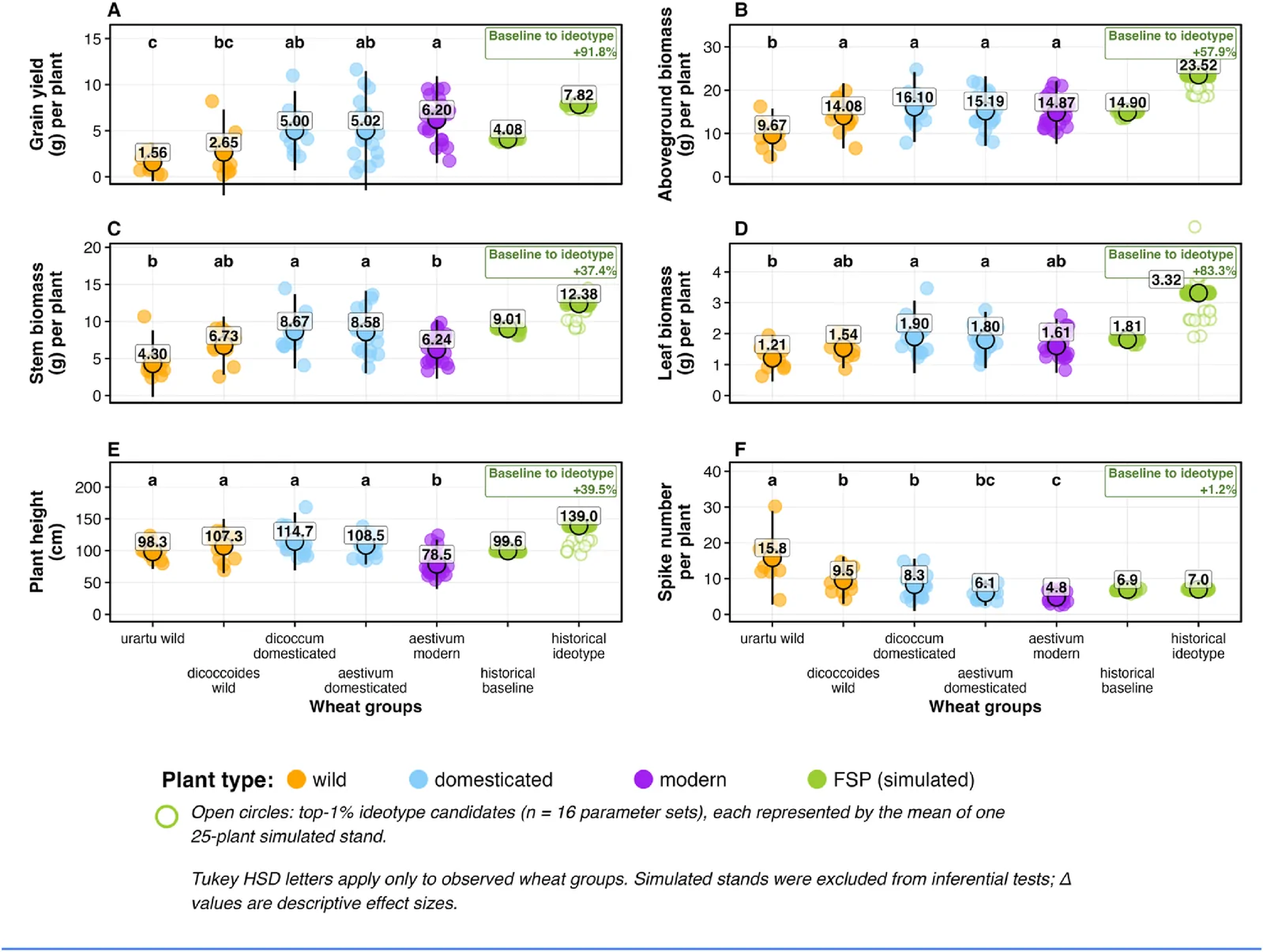
Output trait values for the historical diversity of wheats compared with the ideotype after FSP model simulations from the historical baseline using optimised trait combinations. (A) Grain yield per plant; (B) aboveground biomass per plant; (C) stem biomass per plant; (D) leaf biomass per plant; (E) plant height; and (F) spike number per plant. Points for observed wheat represent individual accessions. Filled green points represent individual plants within one simulated stand and are not independent replicates. Large points and error bars show group means ± SD. Open green circles represent the top 1% ideotype candidates (n = 16 parameter sets), and each circle represents the mean of one 25 plant simulated stand. Percentage changes from the historical baseline to the best ideotype are descriptive effect sizes. Different letters indicate differences among observed wheat groups at P < 0.05 using Tukey HSD. Simulated scenarios and top 1% candidates were excluded from inferential tests. All simulations were carried out at 16 plants/m^2^.

### 3.2 Modern ideotype results

The modern ideotype and the simulations based on all 1598 combinations are shown in Fig. S2B. The morphological diversity of real modern wheat (*T. aestivum*) listed by release dates (1935-2006) is compared with the modern baseline and modern ideotype in Fig. 5. This comparison is based on the model input values, which are the five trait values used for optimization. At a modern planting density of 400 plants/m^2^, modern wheat has achieved the modern ideotype input values for the three biomass traits (Fig. 5A, B and 5C). The potential spike biomass has been increasing over the past seven decades, and the modern varieties with the largest spikes have reached the ideotype optimum (1983 mg) (Fig. 5A; Table S5). Conversely, the potential internode biomass (Fig. 5B) has been gradually decreasing over the past seven decades. However, there is no significant linear relationship between potential leaf biomass and release year. The potential leaf biomass of the modern baseline has also reached the modern ideotype values (Fig. 5C; Table S5; potential leaf biomass = 87 mg). The range encompassing the top 1% modern ideotype candidates also supports these findings, showing an overlap in ranges with observed values for modern wheat (Fig. 5A, B and 5C). The specific internode length and leaf insertion angle of the modern ideotype fall beyond the range of modern wheats (Fig. 5D and E). The specific internode length of modern wheat does not show significant increases and it is still far from the 0.81 mm/mg suggested by modern ideotype (Fig. 5D). The minimum specific internode length of the top 1% modern ideotype candidates far exceeds the maximum value observed in modern wheats (Fig. 5D). The leaf insertion angle (Fig. 5E) of modern wheat has no obvious increase or decrease trend over time, but the modern ideotype suggests increasing it to 35.778° (Fig. 5E). However, the top 1% modern ideotype candidates exhibit a broad range of leaf insertion angles, and a subset of modern wheats already falls within this optimal interval (Fig. 5E).

**Fig. 5:**
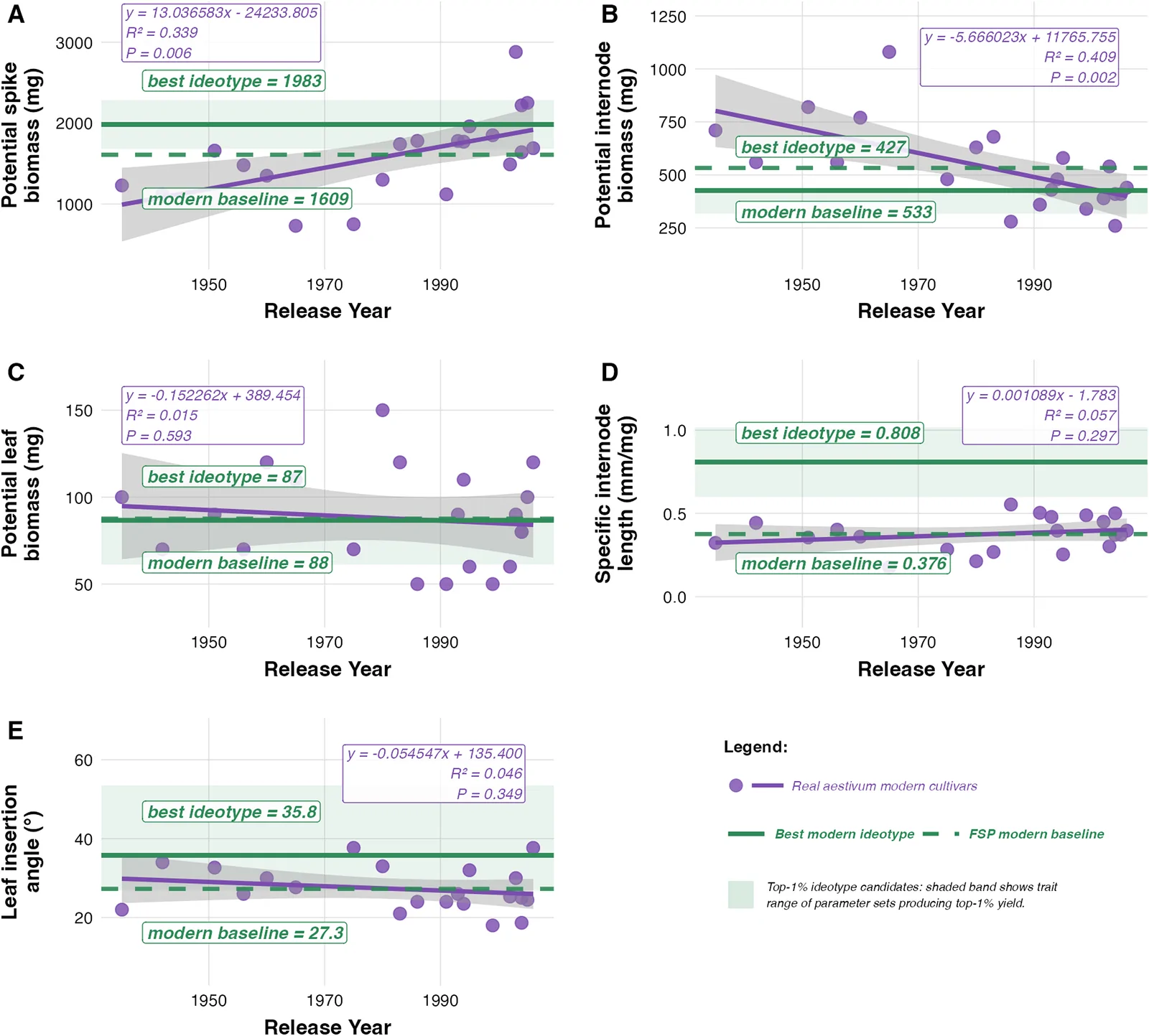
Measured trait values for modern wheats compared with the optimised ideotype values derived from FSP model using the modern baseline. Linear projections of modern *T. aestivum* trait values (purple points) are based on regression against varietal release dates. Green dashed lines indicate the input trait values of the baseline modern wheat model, whereas green solid lines indicate the optimized input trait values of the high density modern wheat ideotype. Panels show biomass traits: (A) potential spike biomass; (B) potential internode biomass; and (C) potential leaf biomass; (D) specific internode length; and (E) leaf insertion angle. The solid purple line is a linear projection from the regression of modern *T.aestivum* values, and the shaded area represents the 95% confidence interval of the linear regression. The trait values of modern model are also the average values of modern wheat traits. The light green shaded band illustrates the trait range of the top 1% ideotype candidates, encompassing all parameter sets that produced a top 1% yield.

Trait optimization significantly enhanced yield and altered plant architecture across both low (16 plants/m^2^) and high (400 plants/m^2^) planting densities (Fig. 6; Fig. S4). The 3D imaging records from the FSP model show that the modern ideotype for wheat is taller than the unoptimized modern baseline, regardless of whether the crop is planted at 16 plants/m^2^ or 400 plants/m^2^ (Fig. 6C; Table S5). At high density, the model frequently generated phenotypes with a wide range of tiller numbers (2–7 tillers per plant), but the highest-yielding solutions consistently converged on about 5 tillers (Fig. 6C; Table S5). While the model reproduces general density-dependent trends, the magnitude of tiller number variation is smaller than typically observed in field-grown wheat. After optimization at 400 plants/m^2^, the height of the modern ideotype reached 126 cm, which is much taller than the modern baseline (Fig. S4D). The aboveground biomass, leaf area and leaf biomass are all increased (Fig. S4B, C and E). However, the stem biomass of the modern ideotype is a little smaller than the modern baseline. The field yield of the modern ideotype at 400 plants/m^2^ has reached 8.828 t/ha, which is higher than that of the modern baseline (5.848 t/ha). In terms of yield per plant, the modern ideotype has a smaller coefficient of variation than the modern baseline (Fig. S4; 0.03 < 0.07). This yield advantage is also retained at 16 plants/m^2^ by the modern ideotype (Fig. 6A). The harvest index of the modern ideotype is also higher than that of the modern baseline, whether at 16 plants/m^2^ or 400 plants/m^2^.

**Fig. 6:**
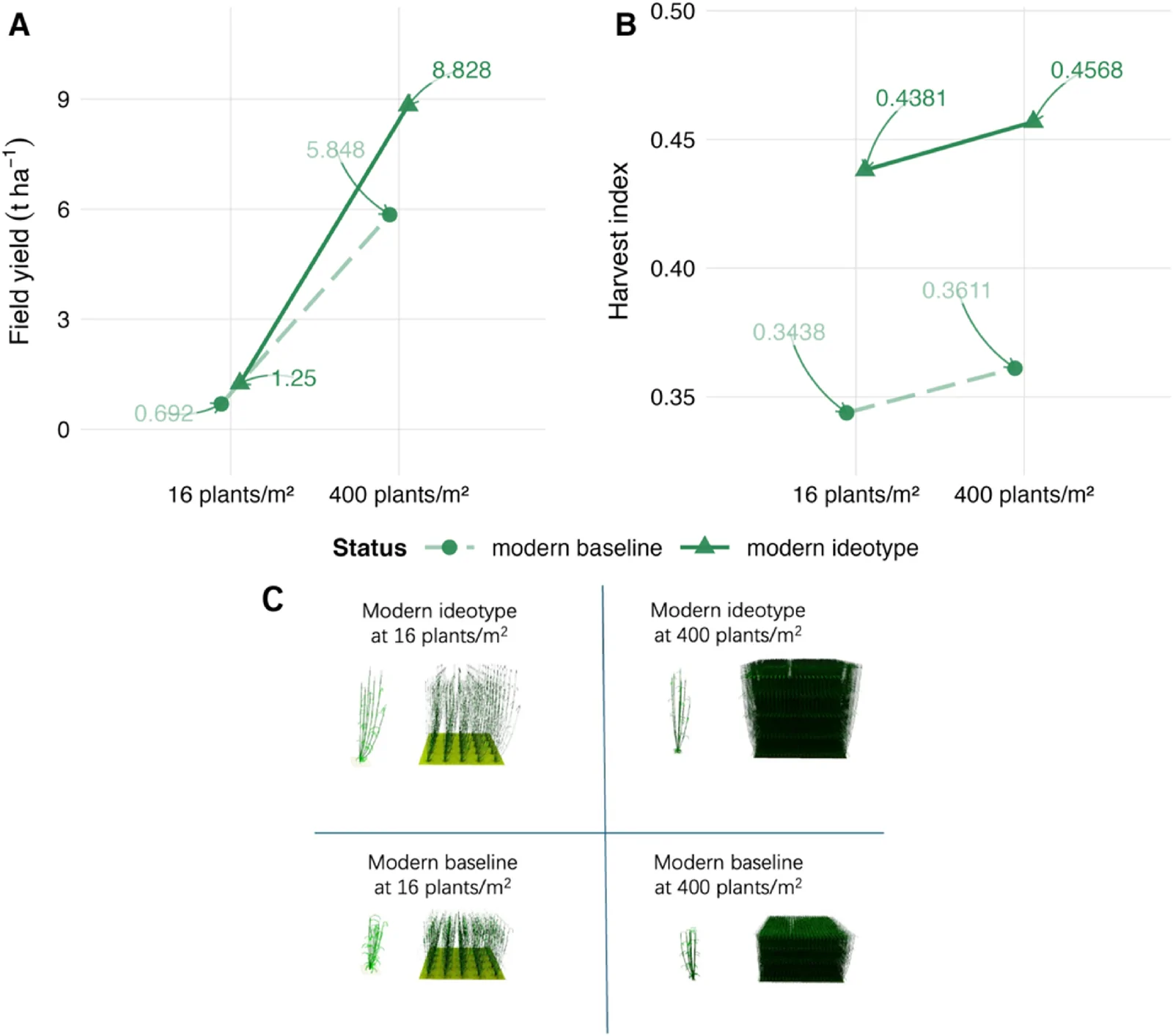
Performance comparison between the baseline modern model and the optimised modern ideotype across low and high planting densities. (A) Simulated field yield (t ha^−1^); and (B) Harvest index. The dashed green line with circles represents the modern model (using the baseline average trait values of modern wheat). The solid green line with triangles represents the modern ideotype (the specific trait combination optimised for maximum yield at 400 plants/m^2^). Both models were simulated at a low density (16 plants/m^2^) and a high density (400 plants/m^2^) to evaluate their performance. Numeric labels adjacent to the data points indicate the exact simulated values. (C) 3D visualisations of the FSP models, illustrating the individual plant architecture (left) and the corresponding crop canopy (right) for each of the four simulated scenarios.

Among the top 1% of modern ideotype candidates (Table S5), potential spike biomass exhibits the largest shift required from the modern baseline to achieve the theoretical yield ceiling (Fig. S5). This analysis only includes data from modern wheat and the modern model. Fig. S5 shows that the potential spike biomass has the largest shift among the five optimised traits. Its shift distribution is highly concentrated in the positive region (median shift approximately 340 mg), indicating that increasing reproductive sink capacity is an important modelling route to higher yield. However, grain yield also depends on fruiting efficiency, defined as grain number per unit of spike dry weight [44]. The current model calculates yield as reproductive organ biomass and does not distinguish grain number, grain mass or other spike components. It therefore cannot evaluate fruiting efficiency. The required shift in potential internode biomass was predominantly negative, with a median of approximately -150 mg and a wide range of variation. The median shift in potential leaf biomass was closed to zero. The offsets of the specific internode length and leaf insertion angle are relatively small and compact, with their distributions close to the zero axis.

## 4 Discussion

By comparing real plant phenotypic evolution with FSP optimised ideotypes for both low-density historical and high-density modern planting conditions, we found that long-term domestication and modern breeding trajectories largely align with theoretical optima. Throughout wheat evolution, traits governing resource sink capacity in potential spike, leaf and internode biomass have consistently converged towards the yield-maximizing values predicted by our models. Furthermore, the analysis of the top 1% ideotype candidates revealed that optimal yields can be achieved through diverse phenotypic synergies rather than a single rigid trait combination. However, plant architectural traits reveal distinct gaps between observed cultivars and idealised phenotypes. The models predicted optimal specific internode lengths and leaf insertion angles that differ from the persistently decreasing trends seen in modern breeding. The ideotype prefers a large specific internode length, leading to tall stems. However, the reason for this is that our current model does not simulate biomechanics and the yield penalties associated with lodging. Therefore, while our analyses highlight potential yield advantages by redesigning modern wheat morphology using natural variation from landraces, these architectural targets reflect mathematical optima that must be balanced against the physical realities of the field.

### 4.1 Reconstructing wheat evolution through the historical ideotype links low density to larger plant size

Our work shows that the phenotypes observed in landraces are largely consistent with yield optimization at low density, mainly in terms of increased potential spike biomass (Fig. 3A), increased potential internode biomass (Fig. 3B), and shorter specific internode length (Fig. 3D). These three traits progressively increased throughout domestication, likely driven by unconscious natural selection or conscious farmer preference for larger phenotypes [17], [45]. Although the Green Revolution introduced a shift towards shorter statures [15], it did not cause a statistically significant reduction in potential internode biomass in our data. The optimal historical ideotype also supports maintaining the current internode biomass rather than reducing it further. Similarly, the historical ideotype favours a higher specific internode length of 0.81 mm/mg, closely aligning with domesticated *T. aestivum* landraces. Furthermore, the substantial plant height (Fig. 4E) and aboveground biomass (Fig. 4B) of the historical ideotype demonstrate that in low density, space-abundant environments, increased plant size enhances yield. Larger plants act as stronger individual competitors, leading to higher individual yields [17]. A key prerequisite for this is maintaining high biomass allocation to the spikes. While the Green Revolution achieved this by reducing plant height to correct historically low spike biomass allocation, our historical model supports an alternative perspective: the Green Revolution traits are fundamentally an adaptation to high density cultivation, sacrificing individual plant size to maximize population-level yield. Otherwise, wheat would likely have evolved toward our historical ideotype, increasing the proportion of spike biomass without reducing internode biomass, resulting in overall gigantism (Fig. 4B and E). The potential leaf biomass (Fig. 3C) and total leaf biomass per plant (Fig. 4D) in simulations suggest that domestication was on the right path to optimal yield improvement, but had still not reached the optimal maximum leaf sizes under certain growing conditions. Since the FSP model is designed around a source-sink conceptualisation of plant growth [46], a larger total leaf area may be required to match the increased sink capacity of larger grains and spikes. However, this relationship is strongly dependent on planting density. At higher densities, increased leaf area may lead to intensified competition for light and resources between plants, such that further increases in leaf biomass do not necessarily translate into yield gains. However, this analysis considers only aboveground allocation. Wheat domestication and breeding have also altered root biomass, architecture and anatomy, with consequences for water and nutrient acquisition [47] [48]. Including these traits in future FSP models would provide a more complete whole plant ideotype.

### 4.2 Reduced internode biomass and increased plant height in the modern ideotype

Our research indicates that the morphology of modern wheat is already close to the ideotype. Over the past few decades, modern wheat breeding has built upon the successful experiences of the Green Revolution, developing larger potential spike biomass and lower potential internode biomass (Fig. 5A and 5B). The modern ideotype selects the same optimal value of an increased specific internode length as that of the historical ideotype (0.81, Fig. 5D), resulting in a taller plant (Fig. S4D). The apparent discrepancy between the lower potential internode biomass in Fig. 5B and the taller phenotype in Fig. 1 reflects the distinction between model inputs and outputs. Potential spike biomass and potential internode biomass are input parameters describing organ sink capacities rather than final biomass values. The higher specific internode length of the modern ideotype (Fig. 5D) produces more stem length per unit biomass. Consequently, total stem biomass was similar in the modern baseline and modern ideotype (2.11 and 2.06 g plant^−1^, respectively; Fig. S4F), whereas grain yield increased from 1.46 to 2.21 g plant^−1^ (Fig. S4A). This seemingly contradicts the dwarfing strategy of the Green Revolution [49]. A reasonable deduction is that the fundamental physiological requirement behind Green Revolution dwarfing was actually to minimize internode biomass. Our modern ideotype theoretically achieves the same outcome by developing 'longer but lighter' stems rather than reducing height. Our experimental data indicates that this optimal specific internode length closely aligns with that of domesticated landraces of *T. aestivum* [19]. Despite this increased height under high planting density, the CV (coefficient of variation) among the 25 simulated individuals did not increase compared to the modern baseline (Fig. S4). Because the FSP models that we use explicitly capture 3D plant-plant interactions [17], intense local competition typically exacerbates phenotypic variation. The stable CV indicates that the model did not predict greater variation among individuals in the taller canopy. However, this result does not establish its mechanical or agronomic feasibility because stem strength, wind and lodging were not simulated. The predicted increase in height should therefore be interpreted as a model outcome associated with greater specific internode length, rather than as a direct breeding recommendation. Furthermore, continuing to decrease plant height may negatively impact yield, since excessive dwarfing impairs water and nutrient transport [50] and weakens competitiveness with weeds. However, attaining the ideotype's extremely low stem biomass in modern wheat appears highly improbable in practice (Fig. 5B and S4F). Mechanically, it is unfeasible to combine such a slender, lightweight stem with a massive spike without incurring severe lodging risks. Thus, engineering a tall, low biomass, yet biomechanically robust stem presents a compelling challenge for future crop breeding.

### 4.3 Larger leaves and increased insertion angles in the ideotype are not under selection at present

Both the historical ideotype and modern ideotype advised increasing leaf size (Figs. 3C, S4C and S4E). The modern ideotype showed that the potential leaf biomass of modern wheat has reached that optimal value (87 mg; Fig. 5C), achieving greater total leaf biomass per plant by adjusting other trait values (Fig. S4E). Larger leaves could benefit photosynthetic source strength and assimilate production, thereby indirectly boosting yield [46]. Despite this general optimization trend, the top 1% high yielding candidates in both ideotypes exhibit considerable phenotypic diversity, with some optimal architectures maintaining a substantially lower potential leaf biomass (Fig. 3C; 5C; Tables S4 and S5). Recent studies show that biomass allocation varies across phenological stages and that the timing of canopy stem elongation is closely associated with grain yield [51] [52]. As phenology was not optimised in this study, our ideotypes represent architectural and allocation optima within the developmental schedule of each baseline model.

A comparable long term evolutionary trend is observed for decreasing leaf insertion angle (the angle with the stem), although recent optimization suggests a transition towards relaxed selection pressure. A smaller leaf insertion angle, resulting in steeper leaves relative to the stem, provides a competitive advantage when paired to larger leaves and could limit the competitive effects of neighbours [17]. Reducing leaf insertion angle was previously shown to be effective in the ideotype canopy design of maize [53]. One possible reason is that upright plant structure may be more conducive to reducing external shade from neighbourhood plants versus self-shading from upper leaves, thereby increasing effective photosynthetic leaf area [54], [55]. Another benefit is that steeper leaves allow deeper light penetration, maximizing whole canopy light-use efficiency [56]. In our simulations, both historical ideotype and modern ideotypes converged on an optimal leaf insertion angle of 35.78° (Fig. 3E; 5E; Table S4 and S5). This angle is drastically smaller than the 71.17° observed in the wild progenitor *T. urartu* (Fig. 3E), confirming that a compact canopy favours yield, although it is actually slightly larger than the current modern wheat average. This implies that while reducing the insertion angle was historically crucial for structural compactness and yield improvement, the current range achieved by modern breeding (26.89°-44.67°) is already sufficient. Consequently, further changes to this specific trait are unlikely to yield substantial agronomic benefits.

### 4.4 Feasibility and biomechanical limitations of model ideotype design

This study successfully validates the feasibility of using FSP models for ideotype design. As demonstrated earlier, the simulated historical ideotype aligns closely with the actual evolutionary trajectory of wheat, with both driving progressive yield improvements. For the modern ideotype, we analysed the trait shifts required to enter the top 1% of modern ideotype candidates (Fig. S5). As a result, the model's two primary recommendations (Fig. 5): increasing potential spike biomass and reducing potential internode biomass, precisely mirror the main breeding targets for high density modern wheat over the past 70 years. This confirms that crop modelling serves as a robust tool for establishing and verifying future breeding objectives.

However, inherent model limitations must be acknowledged. Because the same observations informed model parameterisation, calibration and evaluation, this comparison is an internal evaluation rather than an independent validation. No independent dataset containing the required model inputs and output measurements at a comparable planting density and developmental stage was available. Absolute predictions, particularly grain yield and spike number, should therefore be interpreted cautiously. Independent field data across environments are needed to assess model performance beyond the conditions studied. Our FSP model does not simulate wind and plant lodging characteristics, which influences outcomes. Reduced plant height (dwarfing) was a key trait selected for during the Green Revolution [44], not only to provide resistance to collapse, but also to minimize biomass investment in the stem. Because our model does not currently represent the physical support required for aerial structures, it lacks the yield penalty associated with such tall stems. In addition to the oversized spike and small stem mentioned above, our ideotype also suggests large and steep leaves. In the real growing environment, the structural requirements for large blades may make it difficult to keep them upright. Future model simulations may need to account for mechanical support. Similarly, the purely mathematical nature of the optimization can identify local optima that deviate from conventional agronomic observations. For instance, at a 400 plants/m^2^ density, our modern ideotype maintained a relatively high number of tillers (approximately 5 per plant). Although the model correctly incorporates density-dependent tiller abortion, the algorithm achieved maximum yield by balancing more tillers with smaller individual leaves and spikes, whereas real wheat plants typically adapt to such intense competition by producing fewer tillers [57] with larger organs. Future model simulations may need to account for mechanical support and further physiological constraints to bridge the gap between theoretical trait spaces and field-based realities.

In optimization, the convex hull also limits the optimization to trait combinations represented by the current panel. It therefore excludes combinations that may become feasible through wider use of wild relatives, synthetic wheat, interspecific crossing or gene editing. Candidates near the hull boundary may be useful breeding targets, but their uncommon trait combinations may require targeted parent selection and screening. Future work could expand the phenotypic panel and use genomic prediction to assess the genetic feasibility of combinations outside the current hull. Crop models could then test their performance and trait trade-offs across environments before the search space is expanded.

Moreover, validating the results using multiple analytical methods helps to gain a more comprehensive understanding of the phenotype and yield relationship. In supplementing the assessment of trait importance using random forest regression, we found discrepancies between actual wheat observation data and our historical model simulations (Fig. S3). Specifically, potential internode biomass and specific internode length had the highest importance in the observed data, whereas potential spike and leaf biomass ranked highest in the simulations. These differences highlight that different simulation frameworks inherently capture different aspects of biological mechanisms. Therefore, to effectively address these variations and reliably capture physiological interactions, future research must move beyond the application of single models. For example, barley ideotype design has used eight different models [58]. Integrating multiple crop modelling approaches, such as combining functional structure models with process-based or statistical frameworks, will be a key next step in robustly designing ideotypes.

## 5 Conclusions

Our work compares the yield-oriented FSP model ideotype of wheat with the actual phenotypic evolution of wheat. This comparison was conducted in two stages, first between historical domesticated wheat and historical ideotypes, and secondly between modern wheat and modern ideotypes. Through the first comparison, we found that most traits have historically approached their optimal values during domestication and crop evolution. In the modern ideotype comparison, we found that 20^th^ and 21^st^ century adaptation of modern wheat to high density planting is almost complete, among which potential spike biomass, potential leaf biomass and potential internode biomass have all reached their optimal values during the past 70 years. Although specific internode length and leaf insertion angle diverge from the model's absolute optima, these discrepancies may partly reflect biomechanical and agronomic constraints that are not represented in the current model. Modern breeding has already brought these structural traits into a functionally sufficient range. Our work provides some explanation for the evolution of wheat traits in the past and some insights for the future development of higher yielding varieties.

## Authors' contributions

Y.S., J.E., B.S and C.P.O. designed the experiments. Y.S., J.E., and C.P.O. designed the FSP modeling. Y.S. and B.S. collected and analysed simulation data. Y.S. and C.P.O. wrote the first draft of the manuscript. All authors contributed to data interpretation and revisions to the manuscript.

## Data availability

The code needed to run the FSP model is available from GitHub: https://github.com/YixiangShan/FSP_wheat_domestication. The model data used in this paper can be generated from the model in our repository. The wheat data is from our previous paper [19] and can be download from the DRYAD data repository: http://datadryad.org/dataset/doi:10.5061/dryad.kwh70rzm1.

## Funding

This research was supported by the University of Sheffield and a Chinese Scholarship Council scholarship from the Chinese Government, and the Horizon Europe PV4Plants project (Innovate UK 10049073).

## Declaration of competing interest

The authors declare that they have no known competing financial interests or personal relationships that could have appeared to influence the work reported in this paper.

## Footnotes

Appendix A Supplementary data to this article can be found online at https://doi.org/10.1016/j.plaphe.2026.100290.
