# Supplementary material for: How close have historical domestication and modern breeding come to ideotypes for yield?
Source: Plant Phenomics. 2026 Oct 10;8(4):100290. doi: 10.1016/j.plaphe.2026.100290 (PMC13735239; doi:10.1016/j.plaphe.2026.100290)
Supplement: Supplementary file 1 [file mmc1.pdf]

How close have historical domestication and modern breeding come to ideotypes for yield?

Yixiang Shan[1,2*], Jochem Evers[3], Bo Sun[4], Colin P. Osborne[1*]

[1]Plants, Photosynthesis and Soil, School of Biosciences, University of Sheffield, Sheffield S10 2TN, UK

[2]Institute of Emerging Agricultural Technology, Shenzhen University of Advanced Technology, Shenzhen, Guangdong, China

[3]Centre for Crop Systems Analysis, Wageningen University and Research, Wageningen, The Netherlands

[4]Department of Automatic Control and Systems Engineering, University of Sheffield, Sheffield, UK

Correspondence: Yixiang Shan (shanyixiang1996@gmail.com) & Colin P. Osborne (c.p.osborne@sheffield.ac.uk)

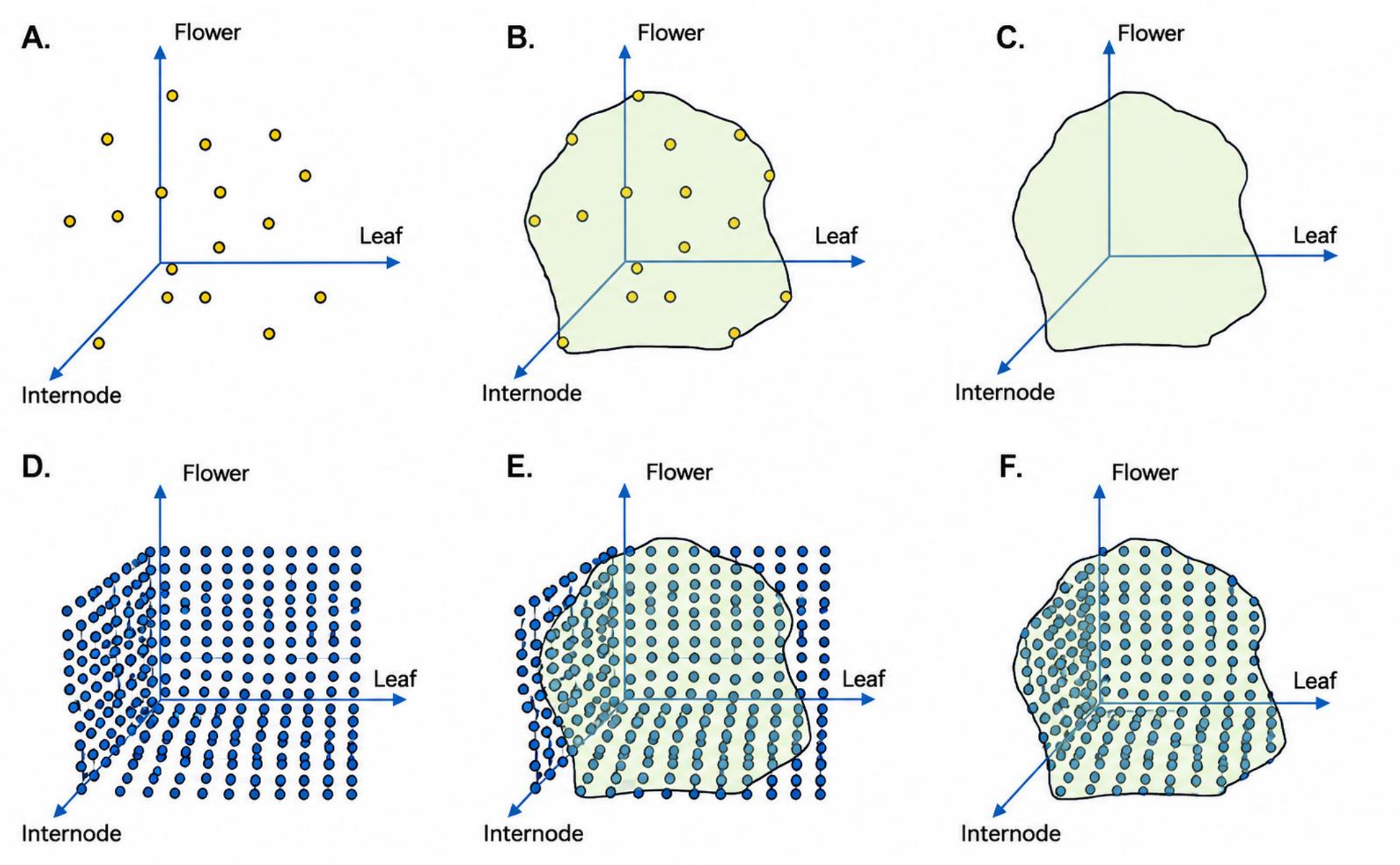

Fig S1. The process of filtering trait combinations via an observation-based convex hull, using three biomass traits as a 3D example. (A) Empirical data points (yellow) representing real plant observations are plotted in a 3D parameter space (Flower, Leaf, and Internode biomass). (B-C) A convex hull (green volume) is constructed around these real observations to define the biologically viable trait boundaries. (D) A uniform 3D grid (blue points) is generated, representing all possible permutations and combinations of the trait values. (E-F) The generated grid is filtered through the convex hull; points falling outside the boundary are discarded, retaining only the trait combinations that exist within the natural phenotypic envelope for subsequent FSP model simulations.

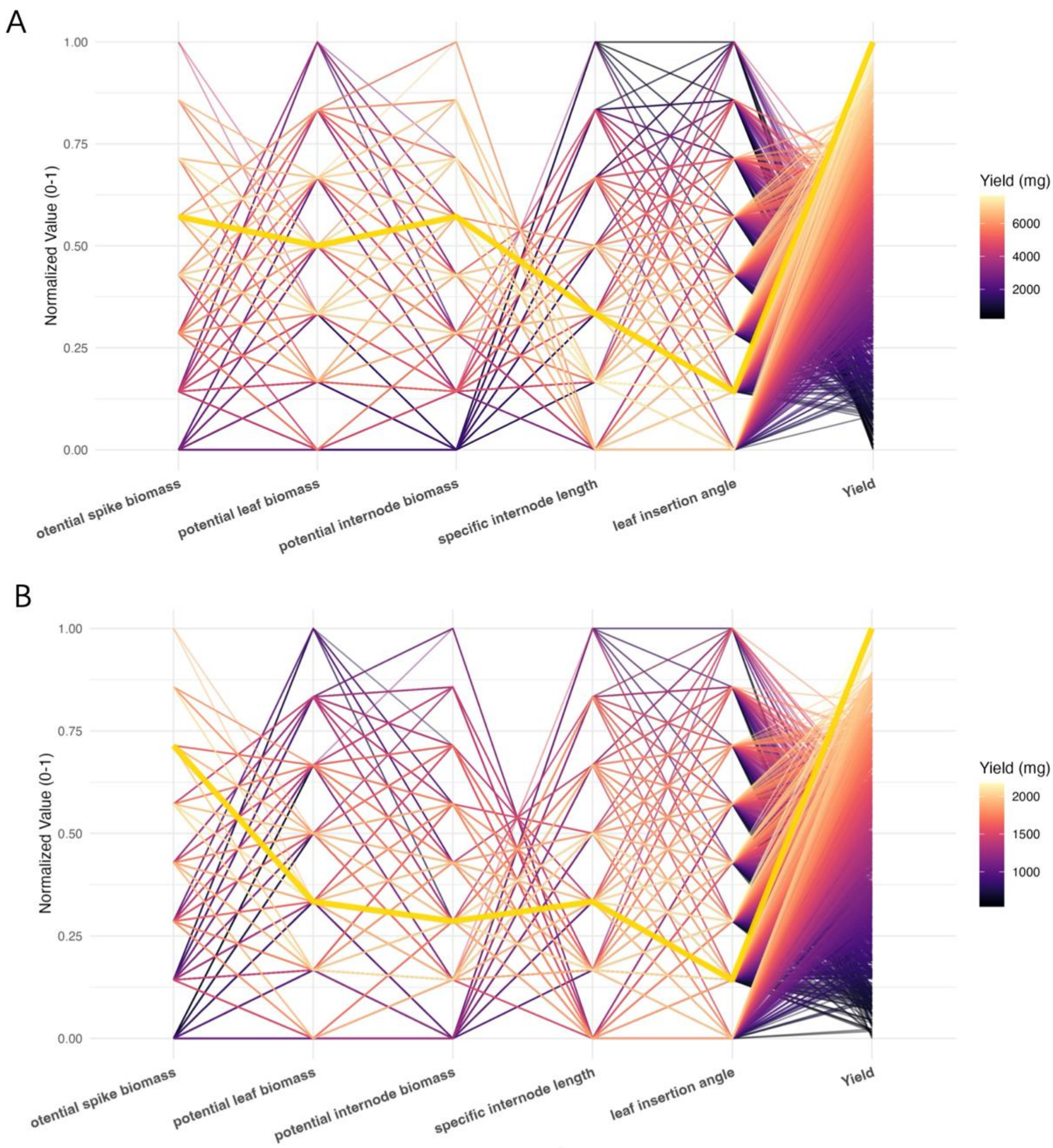

Fig. S2. Parallel coordinates plot characterizing the multi-dimensional phenotypic space and yield performance. (A) historical model and data (B) modern model and data. Each consecutive broken line represents a specific combination of phenotypic traits and all trait values were standardized to the 0-1 interval. The colour is yields of this combination and mapped to the heatmap from low to high. The bold gold line represents the trait combination with the highest yield.

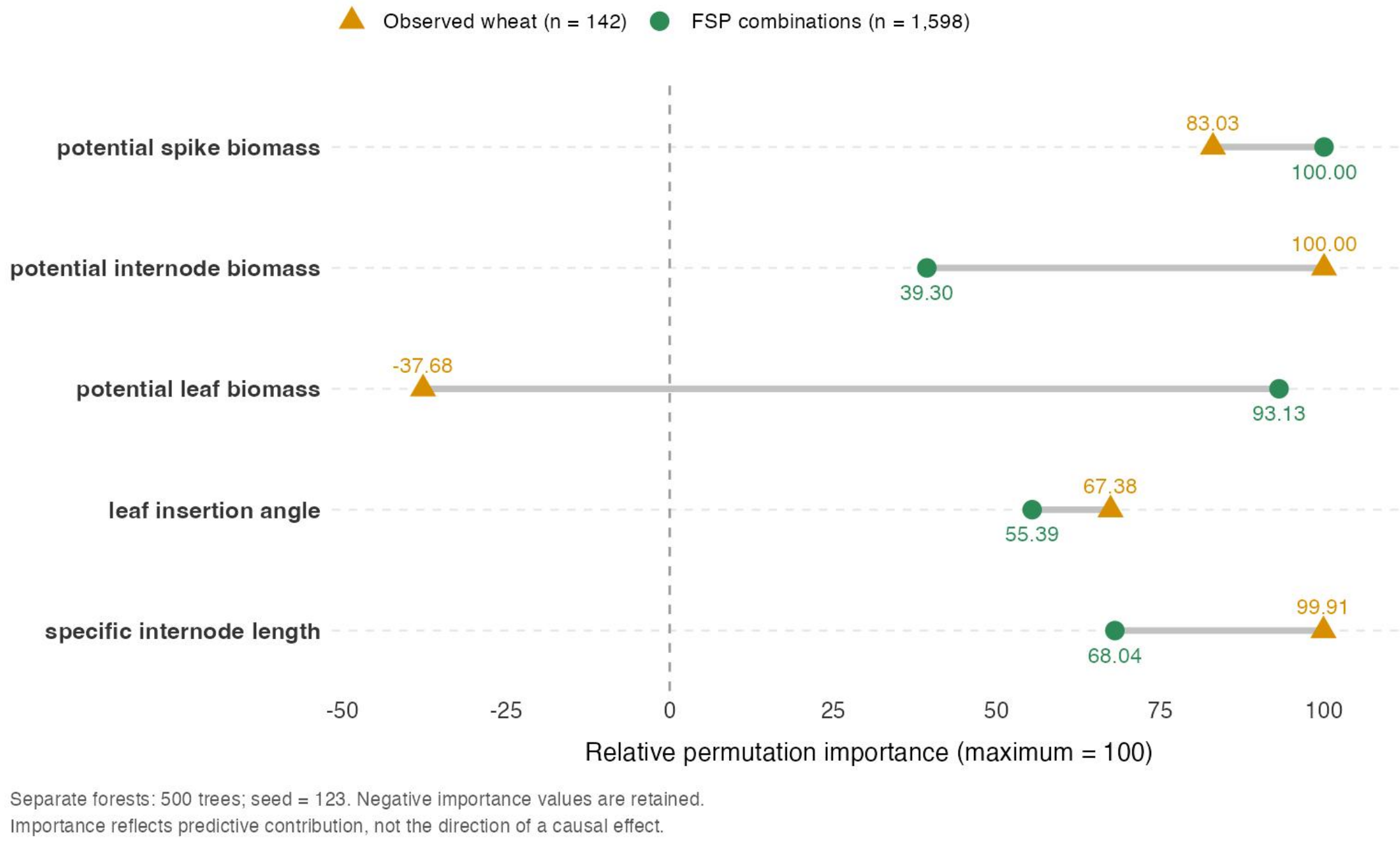

Fig. S3. Comparison of phenotypic trait importance ranking for yield determination between real wheat observations and historical model simulations. Random Forest regression was used to model real wheat datasets (orange dots) and historical simulated datasets (green dots), respectively. Permutation importance was scaled to a maximum of 100 within each dataset, with negative values retained.. The horizontal lines connect the relative importance scores for each trait across the two datasets.

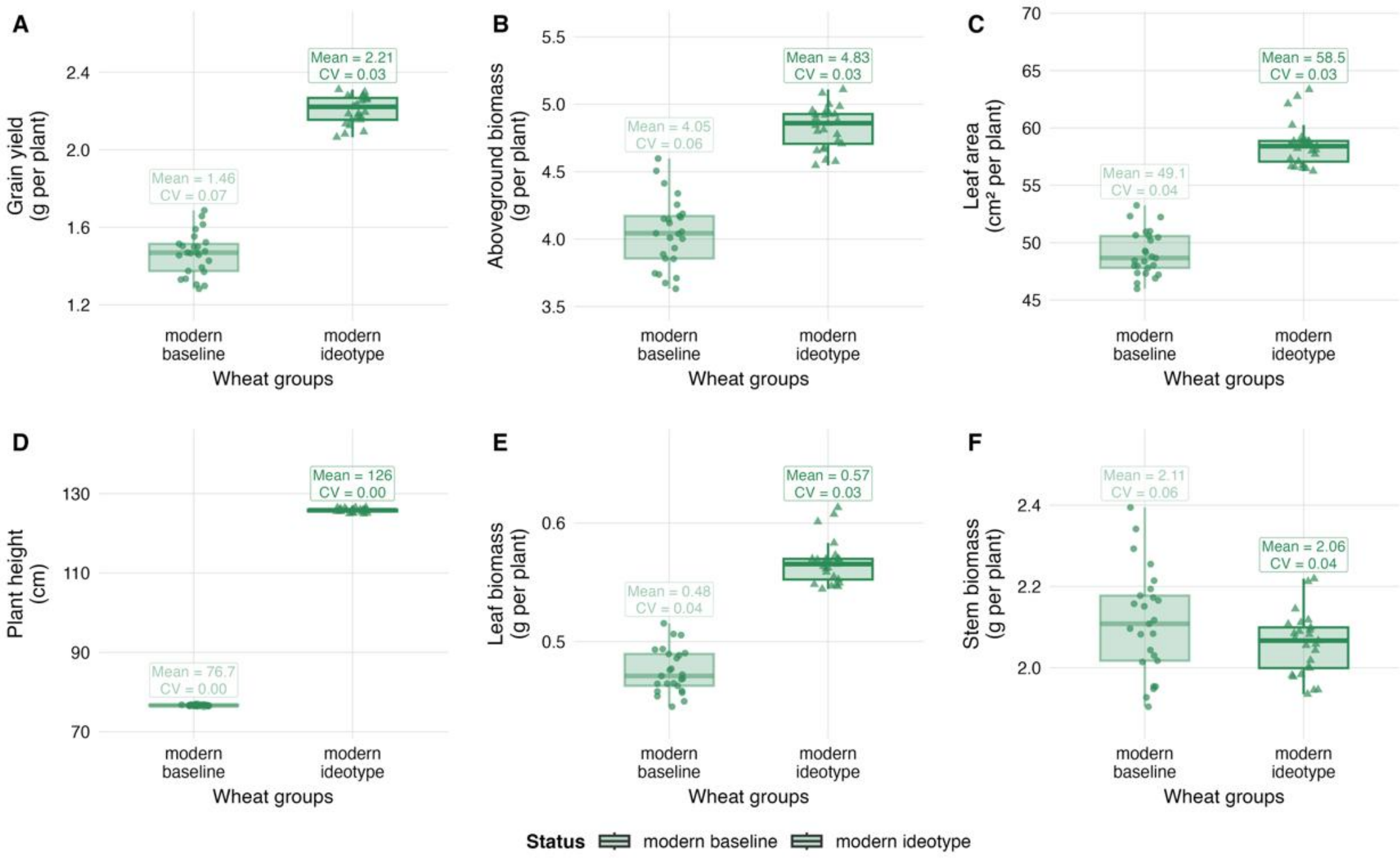

Fig. S4. Comparison of simulated plant-level traits between the modern baseline and modern ideotype under a high planting density (400 plants/m²). (A) grain weight; (B) aboveground biomass; (C) leaf area; (D) plant height; (E) leaf biomass; and (F) stem biomass per individual plant. Boxplots illustrate the data distribution across 25 individual plants within each simulated canopy. Jittered scatter points represent the exact trait values for each individual to visualize within-canopy variation. The mean and coefficient of variation (CV) are annotated for each group. The low CV values in the modern ideotype demonstrate that its superior yield performance is achieved through uniform individual growth, confirming that the optimisation does not result in extreme dominance or suppression among plants under intense high-density competition.

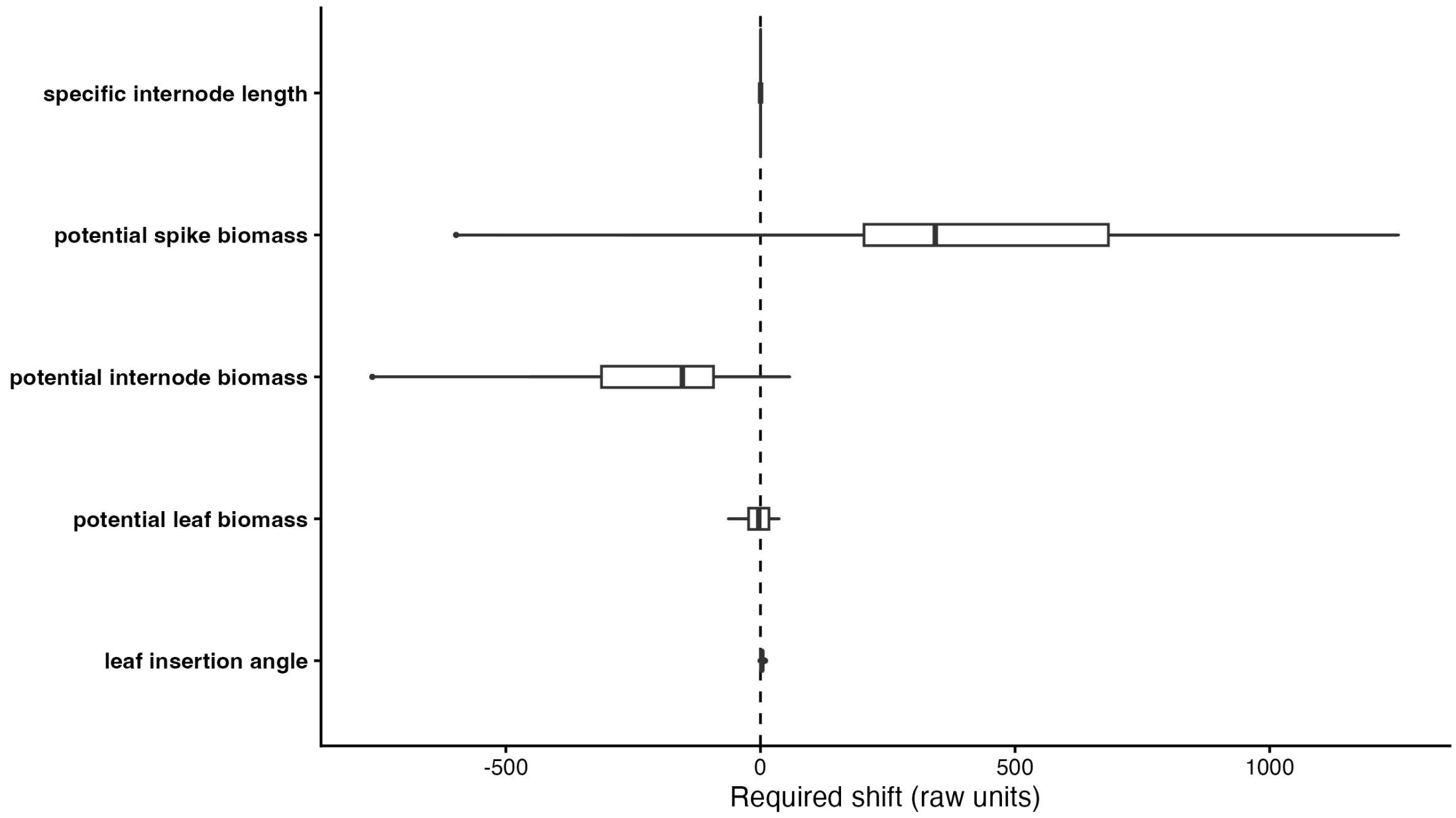

Fig. S5. Distribution of required phenotypic trait shifts to move real-world wheat profiles into the simulated top 1% modern ideotype candidates. Based on the principle of minimizing Euclidean distance, the minimum trait change required for each real-world wheat sample to migrate to the top 1% modern ideotype candidate region predicted by the model within the standardized trait space was calculated.

Table S1. Glossary of terms used in this paper.

**model variable**	**text in paper**	**explanation**	**unit**
wmaxFlower	potential spike biomass	maximum individual flower/fruit biomass	mg
wmaxInt	potential leaf biomass	maximum individual leaf biomass	mg
wmaxLeaf	potential internode biomass	maximum individual internode biomass	mg
specificInternodeLength	specific internode length	internode ratio length / biomass	mm/mg
leafAngleUpper	leaf insertion angle	angle between consecutive leaves along a stem for the lower phytomers	°
leafAngleLower	leaf insertion angle	angle between consecutive leaves along a stem for the upper phytomers	°
yield(mg) in plant output	grain yield per plant	yield per plant	g
Plant height in plant output	plant height	plant height	cm
yield in field output	field yield	biomass of the reproductive organ of the focal plants based on the unit field area	gr/m2
harvestIndex in field output	harvest index	the yield / aboveground plant biomass on the field level	/
aboveBiom in plant output	aboveground biomass	aboveground biomass (leaf+stem+reproductive organs) per plant	g
leafMass in plant output	all leaf biomass	all leaf biomass per plant	g
stemMass in plant output	all stem biomass	all stem biomass per plant	g
nrBranches in plant output	spike number per plant	number of branches on the plant (non-dormant buds)	/
assCO2 in field output	assimilated CO2	canopy assimilated CO2 (umol / m2)	mol/m2

Table S2. Parameters calibrated in the FSP model and method of calculation using measured values from the real wheat experiment.

**Input parameters**	**Corresponding values or calculations in actual experiments**
Delay (days)	[Germination_delay]
rowDistance (m)	Set it as density required; rowDistance = 0.25;
plantDistance (m)	Set it as density required. plantDistance = 0.25;
wmaxFlower (mg)	[One_spike_biomass_July]
wmaxInt (mg)	[Internode_biomass_July]/[Peduncle_biomass_July]
wmaxLeaf (mg)	One_leaf_biomass_July
maxWidthInt (mg)	[Stem_diameter_July]
specificInternodeLength (mm/mg)	[Peduncle_length_July], [Peduncle_biomass_July] -----calculate
lwRatio	[Leaf_length_July], [Leaf_width_July]---------calculate
maxWidth	[Leaf_position_June]
leafCurve (angle)	[Leaf_curtivation_June]
leafAngleLower (angle)	[Leaf_insertation_June]
leafAngleUpper (angle)	[Leaf_insertation_June]
petioleFraction	[Leafpetiole_ratio_harvest]
phyllotaxisLower (angle)	[Leaf_cur_angle_harvest]
phyllotaxisUpper (angle)	[Leaf_cur_angle_harvest]
varDelay (days)	[Germination_delay]
seedMass (mg)	[Seed_weight/Grain_weight]

**Output parameters (plant level)**	**Corresponding values or calculations in actual experiments**
nrBranches	Select the max tiller number from [Tiller_number_June] and [Spike_number_July]
Yield (mg)	[Grain_weight_onespike_hravest], [Spikelet_number_harvest], [Spikelet_number_July], [Spike_number_July], [Spike_number_harvest] -------- calculate expected yielding
leafMass (mg)	[Leaf_mass_July]
stemmas (mg)	[Shoot_mass_July]
aboveBiom (mg)	[Leaf_biomass_July], [Shoot_biomass_July], [Flower_biomass_July] --------- calculate
Plant height (m)	[Plant_height_July]

Table S3. Internal evaluation of historical and modern baseline models at 16 plants/m². Values are means and standard deviations across observed accessions or simulated plants. Mean difference = simulated mean − observed mean. Absolute difference / observed SD = |simulated mean − observed mean| / observed SD. RMSE, MAE and R^2 were not calculated because observed accessions and simulated plants were not paired. Calculating these metrics from arbitrarily paired individuals would not provide a valid measure of prediction error. This is an internal evaluation because the observations also informed model parameterisation and calibration.

Baseline	Output trait	Unit	Observed n	Simulated n	Observed mean	Observed SD	Simulated mean	Simulated SD	Mean difference	Absolute difference / observed SD
Historical	Grain yield per plant	g plant⁻¹	142	25	4.46	3.01	4.08	0.10	−0.38	0.13
Historical	Aboveground biomass	g plant⁻¹	142	25	15.09	4.52	14.90	0.43	−0.19	0.04
Historical	Stem biomass	g plant⁻¹	142	25	7.75	3.02	9.01	0.28	1.26	0.42
Historical	Leaf biomass	g plant⁻¹	142	25	1.68	0.49	1.81	0.05	0.13	0.27
Historical	Plant height	cm	142	25	105.19	23.43	99.65	0.52	−5.54	0.24
Historical	Spike number	plant⁻¹	142	25	8.73	4.66	6.92	0.28	−1.81	0.39
Modern	Grain yield per plant	g plant⁻¹	21	25	6.20	2.37	4.33	0.05	−1.88	0.79
Modern	Aboveground biomass	g plant⁻¹	21	25	14.87	3.63	12.58	0.14	−2.29	0.63
Modern	Stem biomass	g plant⁻¹	21	25	6.24	1.99	7.06	0.08	0.82	0.41
Modern	Leaf biomass	g plant⁻¹	21	25	1.61	0.43	1.20	0.01	−0.41	0.94

Baseline	Output trait	Unit	Observed n	Simulated n	Observed mean	Observed SD	Simulated mean	Simulated SD	Mean difference	Absolute difference / observed SD
Modern	Plant height	cm	21	25	78.55	19.41	77.42	0.28	−1.13	0.06
Modern	Spike number	$plant^{-1}$	21	25	4.81	1.29	6.00	0.00	1.19	0.92

Table S4. Parameters and simulated outputs for the historical model and top 1% ideotype candidates at 16 plants/m². Row 1 shows empirical trait values and measured outputs for real historical wheat. Row 2 displays the simulated outputs for the baseline historical model. Subsequent rows list the top 1% historical ideotype candidates ranked by descending grain yield per plant, starting with the single highest-yielding optimal ideotype (ID 513).

Wheat group	Sim ID	Grain yield (g/plant)	Potential spike biomass (mg)	Potential leaf biomass (mg)	Potential internode biomass (mg)	Specific internode length (m/g)	Leaf angle (°)	Aboveground biomass (g/plant)	Stem biomass (g/plant)	Leaf biomass (g/plant)	Plant height (cm)	Spike number per plant
historical wheat	wheat	4.457	1009	90	455	0.622	40.147	15.090	7.750	1.678	105.189	8.73
historical baseline	model	4.078	1009	90	455	0.622	40.147	14.897	9.010	1.809	99.647	6.92
Top 1% historical ideotype	513	7.822	1684	138	536	0.808	35.778	23.515	12.378	3.316	139.032	7
Top 1% historical ideotype	181	7.690	1983	138	536	0.598	26.889	20.648	10.211	2.748	106.932	7
Top 1% historical ideotype	543	7.665	1684	163	536	0.598	35.778	23.700	12.136	3.899	98.236	7
Top 1% historical ideotype	496	7.655	1983	138	536	0.598	35.778	20.550	10.161	2.734	107.138	7
Top 1% historical ideotype	848	7.597	1983	138	536	0.598	44.667	20.392	10.079	2.716	107.193	7
Top 1% historical ideotype	892	7.570	1684	163	536	0.598	44.667	23.386	11.975	3.842	98.863	7
Top 1% historical ideotype	376	7.457	1684	87	427	1.019	35.778	18.736	9.350	1.929	151.022	6.92
Top 1% historical ideotype	560	7.425	1386	189	536	0.598	35.778	27.360	14.479	5.456	94.002	7.04
Top 1% historical ideotype	735	7.345	1684	87	427	1.019	44.667	18.431	9.188	1.898	150.979	6.88
Top 1% historical ideotype	208	7.321	1684	163	644	0.598	26.889	24.893	13.875	3.697	121.356	7
Top 1% historical ideotype	547	7.318	1684	163	644	0.598	35.778	24.886	13.868	3.700	121.168	7
Top 1% historical ideotype	151	7.286	1684	112	427	0.808	26.889	18.885	9.137	2.461	116.052	7
Top 1% historical ideotype	442	7.277	1684	112	427	0.808	35.778	18.859	9.123	2.459	116.031	7
Top 1% historical ideotype	447	7.260	1684	112	536	0.808	35.778	21.130	11.422	2.448	147.306	7
Top 1% historical ideotype	799	7.212	1684	112	427	0.808	44.667	18.685	9.038	2.435	116.304	7
Top 1% historical ideotype	804	7.206	1684	112	536	0.808	44.667	20.962	11.329	2.427	147.851	7

Table S5. Parameters and simulated outputs for the modern model and top 1% ideotype candidates at 400 plants/m². Row 1 shows empirical input traits for real modern wheat; output variables are omitted (/) because empirical data were collected at 16 plants/m² and cannot be directly compared. Row 2 displays the simulated outputs for the baseline modern model. Subsequent rows list the top 1% ideotype candidates ranked by descending grain yield per plant, starting with the single highest yielding optimal ideotype

Wheat group	ID	Grain yield (g/plant)	Potential spike biomass (mg)	Potential leaf biomass (mg)	Potential internode biomass (mg)	Specific internode length (mm/mg)	Leaf angle (°)	Leaf area (cm²)	Aboveground biomass (g/plant)	Stem biomass (g/plant)	Leaf biomass (g/plant)	Plant height (cm)	Spike number per plant
modern wheat	wheat	/	1609	87.6	533	0.376	27.270	49.089	/	/	/	/	/
modern baseline	model	1.462	1609	87.6	533	0.376	27.270	49.089	4.049	2.112	0.475	76.656	5
Top 1% modern ideotype	358	2.199	1983	87	427	0.808	35.778	57.990	4.798	2.037	0.561	125.757	5
Top 1% modern ideotype	716	2.179	1983	87	427	0.808	44.667	57.529	4.755	2.020	0.557	125.956	5
Top 1% modern ideotype	1034	2.177	1983	87	427	0.808	53.556	57.235	4.746	2.016	0.554	126.113	5
Top 1% modern ideotype	376	2.174	1684	87	427	1.019	35.778	68.927	5.345	2.504	0.667	155.674	6
Top 1% modern ideotype	735	2.152	1684	87	427	1.019	44.667	68.556	5.304	2.488	0.664	156.048	6
Top 1% modern ideotype	334	2.108	2282	87	427	0.598	35.778	51.112	4.316	1.713	0.495	93.412	5
Top 1% modern ideotype	692	2.108	2282	87	427	0.598	44.667	50.832	4.310	1.710	0.492	93.448	5
Top 1% modern ideotype	280	2.090	1684	61	318	1.019	35.778	39.555	4.075	1.602	0.383	118.549	4.04
Top 1% modern ideotype	638	2.084	1684	61	318	1.019	44.667	38.975	4.044	1.583	0.377	118.701	4
Top 1% modern ideotype	969	2.076	1684	61	318	1.019	53.556	38.469	4.022	1.573	0.372	118.981	4
Top 1% modern ideotype	73	2.005	1983	87	318	0.598	26.889	55.593	3.959	1.416	0.538	69.244	5.04
Top 1% modern ideotype	327	2.004	1983	87	318	0.598	35.778	55.327	3.949	1.409	0.536	69.279	5
Top 1% modern ideotype	352	2.002	1684	87	318	0.808	35.778	65.878	4.383	1.743	0.638	92.257	5.88
Top 1% modern ideotype	94	2.001	1684	87	318	0.808	26.889	65.920	4.382	1.743	0.638	92.212	5.88
Top 1% modern ideotype	710	2.000	1684	87	318	0.808	44.667	65.470	4.369	1.736	0.634	92.398	5.84

Top 1% modern ideotype	715	1.999	1684	87	427	0.808	44.667	61.705	4.798	2.202	0.597	125.150	5.32
